# RNA modification in cardiovascular disease: implications for therapeutic interventions

**DOI:** 10.1038/s41392-023-01638-7

**Published:** 2023-10-27

**Authors:** Cong Wang, Xuyang Hou, Qing Guan, Huiling Zhou, Li Zhou, Lijun Liu, Jijia Liu, Feng Li, Wei Li, Haidan Liu

**Affiliations:** 1 Department of Cardiovascular Surgery, The Second Xiangya Hospital of Central South University, Changsha, Hunan China; 2 Clinical Center for Gene Diagnosis and Therapy, The Second Xiangya Hospital of Central South University, Changsha, Hunan China; 3 Department of Pathology, National Clinical Research Center for Geriatric Disorders, The Xiangya Hospital of Central South University, Changsha, Hunan China; 4 Department of Radiology, The Third Xiangya Hospital of Central South University, Changsha, Hunan China

**Keywords:** Cardiology, Epigenetics

## Abstract

Cardiovascular disease (CVD) is the leading cause of death in the world, with a high incidence and a youth-oriented tendency. RNA modification is ubiquitous and indispensable in cell, maintaining cell homeostasis and function by dynamically regulating gene expression. Accumulating evidence has revealed the role of aberrant gene expression in CVD caused by dysregulated RNA modification. In this review, we focus on nine common RNA modifications: N^6^-methyladenosine (m^6^A), N^1^-methyladenosine (m^1^A), 5-methylcytosine (m^5^C), N^7^-methylguanosine (m^7^G), N^4^-acetylcytosine (ac^4^C), pseudouridine (Ψ), uridylation, adenosine-to-inosine (A-to-I) RNA editing, and modifications of U34 on tRNA wobble. We summarize the key regulators of RNA modification and their effects on gene expression, such as RNA splicing, maturation, transport, stability, and translation. Then, based on the classification of CVD, the mechanisms by which the disease occurs and progresses through RNA modifications are discussed. Potential therapeutic strategies, such as gene therapy, are reviewed based on these mechanisms. Herein, some of the CVD (such as stroke and peripheral vascular disease) are not included due to the limited availability of literature. Finally, the prospective applications and challenges of RNA modification in CVD are discussed for the purpose of facilitating clinical translation. Moreover, we look forward to more studies exploring the mechanisms and roles of RNA modification in CVD in the future, as there are substantial uncultivated areas to be explored.

## Introduction

Dynamic chemical modifications are prevalent in biological macromolecules, particularly RNA and protein, determining the fate of these molecules and regulating their function. For instance, demethylation of DNA can lead to chromatin reprogramming and gene transcription.^1^ The phosphorylation of a protein is crucial to its ubiquitination and degradation by the proteasome.^2^ Over the past half century, many RNA modifications have been thoroughly documented. Numerous studies have confirmed that RNA modification plays an indispensable role in physiologic processes and pathologic changes in mammals. RNA stimulates the mammalian innate immune system by activating toll-like receptors (TLRs), which is significantly inhibited by the incorporation of modified nucleosides 5-methylcytidine (m^5^C), N^6^-methyladenosine (m^6^A), 5-methyluridine (m^5^U), 2-thiouridine (s^2^U), or pseudouridine (denoted by psi, Ψ).^3^ m^6^A is involved in mammalian cortical neurogenesis by promoting the decay of mRNA associated with neurogenesis and neuronal differentiation.^4^ Ψ-mediated post-transcriptional modification of tRNA leads to translation dysregulation, which affects stem cell commitment during early embryogenesis, and is commonly seen in aggressive subtypes of human myelodysplastic syndromes.^5^ Recent studies indicate an association between RNA modification and therapeutic tolerance. Rapino et al.^6^ found that enzymes catalyzing wobble uridine 34 (U34) tRNA modification are responsible for the transformation driven by BRAF V600E oncogene and resistance to targeted therapy of melanoma.

The role of RNA modification in disease is gradually being revealed, providing potential small molecules, and new targets for drug development and intervention strategies (Supplementary Table 1). N^1^-methyladenosine (m^1^A)-modified early tRNAs improve the translation efficiency of MYC protein and promote T cell proliferation, and interventions based on this mechanism can alleviate colitis in mouse model.^7^ Yankova et al.^8^ reported a highly selective m^6^A “writer” inhibitor STM2457. Their study demonstrates that STM2457 acts as an anti-cancer agent by inhibiting METTL3, resulting in the selective reduction of m^6^A levels on the mRNA of known leukemia genes with reduced translation. Delaunay et al.^9^ revealed that some antibiotics could be used as adjuvants in cancer therapy to inhibit metastasis by targeting m^5^C in mitochondrial tRNAs (mt-tRNAs). Combined blocking of N^7^-methylguanosine (m^7^G) tRNA methyltransferase METTL1 and its downstream chemokine pathway can enhance the efficacy of anti-PD-1 in the treatment of intrahepatic cholangiocarcinoma, providing a new idea for developing effective immunotherapy.^10^ In the tremendous progress made in the chemical modification of biomacromolecules, we have seen several drugs move from clinical trials to approval to benefit patients.^11^ Drugs based on RNA modification are in clinical trials,^12^ and we expect new and improved results from them.

**Fig. 1:**
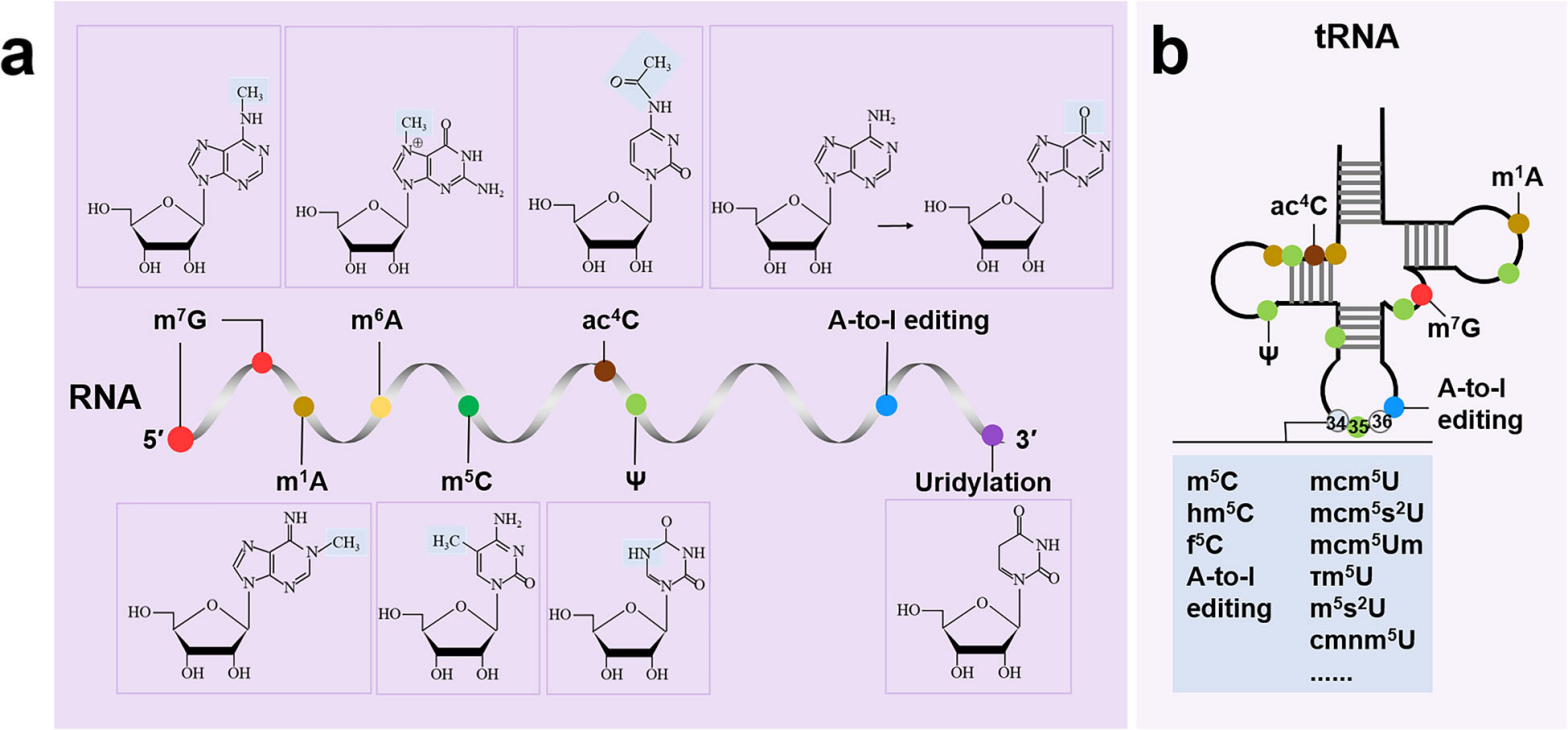
A schematic diagram of chemical modifications on mammal RNAs. Several RNA modifications with their chemical structures are highlighted in (**a**) transcripts (such as mRNA, miRNA and lncRNA) and (**b**) tRNAs. For tRNAs, anticodons are located at positions 34 to 36, and modification at position 34 (known as U34 modification) regulates wobble pairing, including m^5^C, hm^5^C, f^5^C, A-to-I editing, mcm^5^U, mcm^5^s^2^U, mcm^5^Um, τm^5^U, τm^5^s^2^U, and cmnm^5^U. m^7^G, 7-methylguanosine; m^1^A, *N*^1^-methyladenosine; m^6^A, *N*^6^-methyladenosine; m^5^C, 5-methylcytosine; ac^4^C, *N*^4^-acetylcytidine; Ψ, pseudouridine; A-to-I editing, adenosine-to-inosine RNA editing; mcm^5^U, 5-methoxycarbonylmethyluridine; mcm^5^s^2^U, 5-methoxycarbonylmethyl-2-thiouridine; mcm^5^Um, 5-methoxycarbonylmethyl-2’-O-methyluridine; τm^5^U, 5-taurinomethyluridine; τm^5^s^2^U, 5-taurinomethyl-2-thiouridine; cmnm^5^U, 5-carboxyaminomethyluridine

In this review, we mainly focus on nine RNA modifications (Fig. 1) and their effects on cardiovascular diseases, ranging from the development and physiological function of the cardiovascular system to the occurrence and progression of the disease, and the potential therapeutic strategies.

## Overview of RNA modifications

**Fig. 2:**
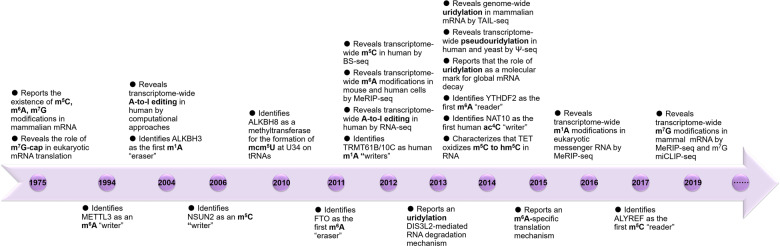
The historical milestone in RNA modification

The development of various scientific and technical methods (e.g., liquid chromatography/mass spectrometry and high-throughput sequencing) has promoted the progression of RNA modifications (Fig. 2; for details on technologies for mapping modified nucleotides, see the review by Bartee et al.^13^ and Wiener et al.^14^). Currently, over 100 RNA modifications have been identified and characterized on various types of RNAs, including messenger RNAs (mRNAs) and non-coding RNAs (ncRNAs), such as ribosomal RNAs (rRNAs) and transfer RNAs (tRNAs), some of them are known to have specific biochemical and physiological effects, and indispensable roles in human diseases.^15^

RNA modification is a dynamic and reversible process theoretically regulated by certain proteins or complexes. The “writers” are responsible for installing modification molecules that can be removed by the “erasers”, which are usually pairs of enzymes with opposite functions, such as methyltransferase and demethylase. In addition, modification sites can be recognized and bound by several proteins, called “readers”, thus regulating RNA metabolism or function. Most “writers”, “erasers”, and “readers” are identified as m^6^A regulators, while regulators in other modifications are reported less frequently.

RNA modification determines or affects RNA fate and function. 5′-cap modification and the poly(A) tail are common structures found in almost every eukaryotic mRNA, which control their stability and facilitate translation. 5′-cap is the m^7^G modified guanine nucleotide, located in the 5′-end of eukaryotic mRNA, while poly(A) tail is ~200 adenosine residues, added to the 3′-end of mRNA in the process of transcription, and is associated with export to the cytoplasm. Moreover, some internal modifications (including but not limited to m^6^A, m^1^A, m^5^C, 2′-O-methylation) on mRNA have recently received more attention. These internal modifications can also be found in various ncRNAs, such as microRNAs (miRNAs) and long non-coding RNAs (lncRNAs). Regarding function, m^6^A modification affects the processing, structure, splicing, localization, stability, degradation, and translation of RNAs and their functions, such as RNA–protein and RNA–RNA interactions.^16–18^ The m^5^C can perform a similar role.^19–22^ Recently, m^1^A and ac^4^C have been found to promote the stability and translation of methylated mRNA.^23–25^ Ψ, a more common modification in ncRNAs, stabilizes the structure of tRNAs and rRNAs and regulates mRNAs in response to environmental signals in humans and yeast.^26^

tRNA is a linking molecule carrying amino acids and anticodons, with abundant modification sites for various modifications (including but not limited to: m^1^A, m^5^C, m^7^G, ac^4^C, and Ψ), which plays an indispensable role in the accurate translation of proteins. For example, the T/D arm and variable loop modifications are normally associated with structural stability of tRNA,^27–29^ while anticodon loop modifications are involved in accurate translation.^30^ Aberrant modifications of cytoplasmic tRNA and mt-tRNA can cause various diseases, such as neurological disorders and tumors. Suzuki summarized the aberrant modifications of tRNAs and the corresponding regulatory genes in these diseases.^31^

The ribosome comprises two subunits containing rRNAs and ribosomal proteins (RPs): the small subunit (SSU) identifying mRNAs and the large subunit (LSU) carrying peptidyl transferase center, translation factor binding sites, and exit tunnel. During the biogenesis of ribosomes, some rRNAs are mainly modified with methylated sugars and Ψs at the subunit inner cores and the interface. How these modifications occur and their effects on rRNA structure and function are discussed by Sharma and Lafontaine.^32^

In addition, with the revelation of functions of mRNAs, lncRNAs and circulating RNAs (circRNAs) in human health and diseases were previously considered junk without biological function. Their interaction with chemical modifications that regulate the transcription, stability, export, and function of these ncRNAs is also beginning to be revealed. For instance, m^6^A modification promotes THAP7-AS1 transcription,^33^ DIAPH1-AS1 stability,^34^ and circNSUN2 export.^35^ Wang et al.^36^ found that oxidative modification confers miR-184 to target Bcl-xL and Bcl-w, inhibiting their translation and promoting apoptosis. The addition of terminal uridine to the 3′-end of miR-26 eliminates its inhibition of IL-6.^37^ Moreover, these ncRNAs, in turn, can regulate chemical modification. LncRNA can serve as a decoy to inhibit histone methyltransferase,^38^ and a scaffold of histone modification complexes to regulate histone modifications on target genes.^39,40^

## The mechanisms of RNA modifications

### N^6^-methyladenosine (m^6^A)

**Fig. 3:**
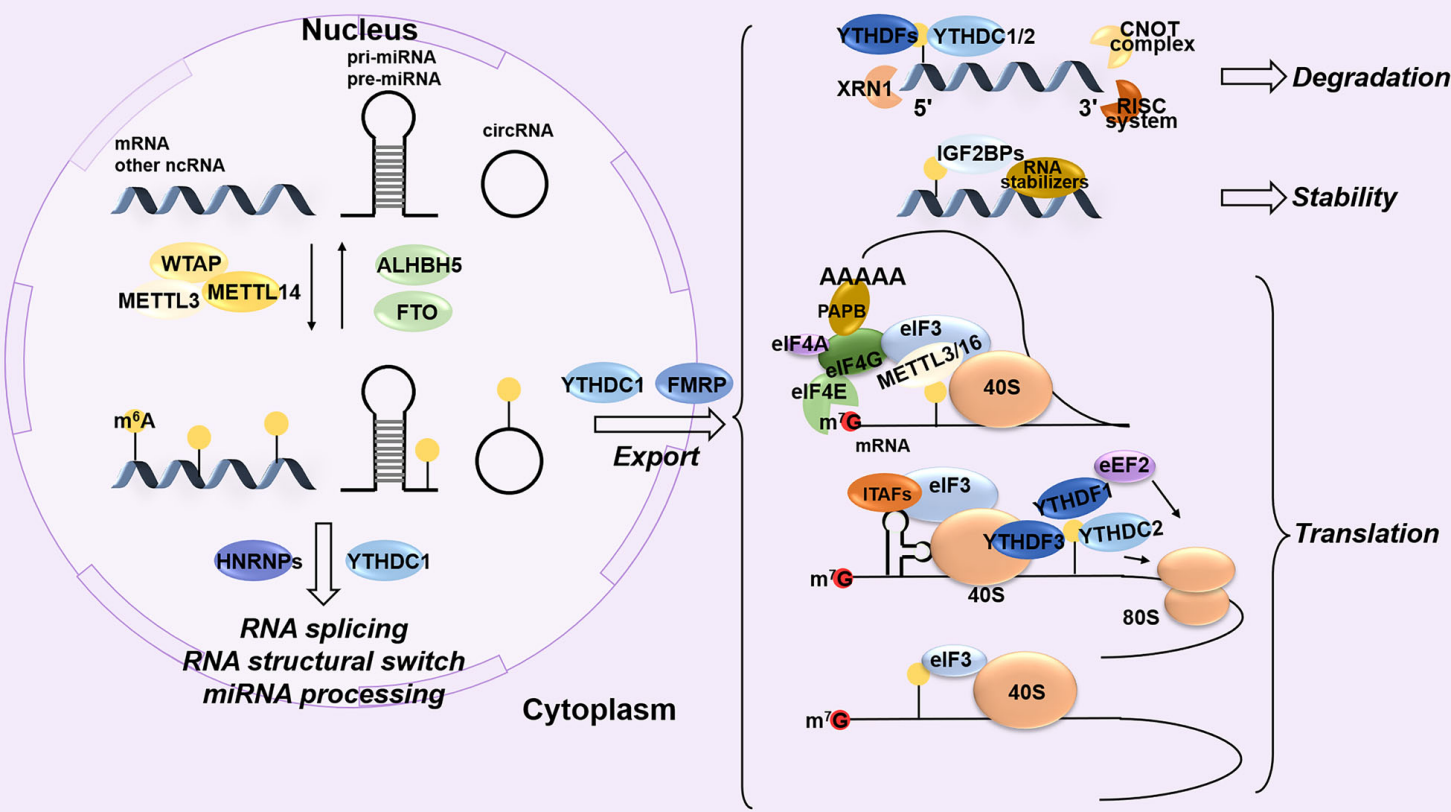
Biological functions of dynamic m^6^A modification on RNAs. m^6^A modification is a reversible process in which “writers” (such as METTL3 and METTL14) add m^6^A to RNAs and “erasers” (ALKBH5 and FTO) are responsible for removal in the nucleus. The “readers” recognize and bind to m^6^A-RNA, which affects RNA processing and export to the cytoplasm. In the cytoplasm, m^6^A finally regulates RNA degradation, stability and translation

Nucleotide methylation in HeLa cells, including m^6^A and m^7^G, was identified about 50 years ago,^41,42^ and has been studied extensively since then. To date, m^6^A is believed to be the most common internal modification of eukaryotic mRNA. However, due to the limitations of the technical methods at the time, much was still unknown in this field. By 2012, Dominissini et al.^43^ used a novel technique, m^6^A-seq (also called MeRIP-seq), to identify transcriptome-wide m^6^A modifications in humans and mice. Their results suggest that m^6^A, which is mainly found around the stop codons and in the longer internal exons, plays an essential regulatory role in gene expression. Using the same technique, Meyer et al.^44^ obtained similar results in mammals that m^6^A sites are enriched near the stop codons, and in 3′-untranslated regions (3'UTRs) which may be related to miRNA-binding sites. The m^6^A mapping and measure techniques, and their advantages and disadvantages, are comprehensively summarized elsewhere (see ref. ^45^).

m^6^A is added to specific sites of RNAs by a multi-subunit complex, methyltransferase-like 3 (METTL3)-METTL14, which is stable in cells.^46^ METTL3 (used to be MT-A70) has enzymatic activity,^47^ and its allosteric homolog METTL14 with higher methyltransferase activity^48^ is critical for target recognition and binding.^49,50^ Wilms’ tumor 1-associating protein (WTAP), without catalytic domains, is identified as a key subunit in the m^6^A methyltransferase complex, which can interact with METTL3 and METTL14 to affect their activity and accumulation in nuclear speckles markedly.^46,48^ It is worth mentioning that the effect of METTL14 and WTAP on m^6^A level in cells is greater than that of METTL3.^46^ In addition, METTL16, VIRMA (also known as KIAA1429), HAKAI (also called CBLL1), ZC3H13, RBM15, RBM15B, METTL5-TRMT112, and ZCCHC4 are identified as m^6^A “writers” in mammals.^51–54^ METTL5-TRMT112 and ZCCHC4 are responsible for m^6^A modification of 18S rRNA and 28S rRNA, respectively.^54^ Moreover, methyltransferases are sequence-specific with site preference rather than structural preference for the target RNA.^46,52^ METTL3 and WTAP bind to a variety of types of RNAs, but most of them are mRNA. Their binding sites are mainly located in the coding sequence (CDS) and 3′UTR.^48^ GGAC and GACU are the main enrichment binding motifs of METTL3-METTL14 and WTAP, respectively.^46^ VIRMA-mediated m^6^A on mRNA is concentrated in the 3′UTR and close to the stop codon, particularly with the GGACU motif.^52^ RRACH (R = G/A; H = A/C/U) is a consensus-specific methylated site with highly conservation.^43,44,55–57^ Demethylase converts the m^6^A into A in RNAs, called the m^6^A “eraser”. Fat mass and obesity-associated protein (FTO) and ALKBH5, members of the AlkB family, are currently reported as m^6^A “erasers” in mammals.^58,59^ In the dynamic process of m^6^A modification, there is a class of proteins, known as m^6^A “readers”, which recognize and bind the m^6^A sites of RNAs, participate in the molecular mechanisms, and mediate important functions. At present, proteins are identified as m^6^A “readers” in mammals, including but not limited to YTH domain family proteins (YTHDF1-3), YTH domain-containing proteins (YTHDC1-2), heterogeneous nuclear ribonucleoprotein C (HNRNPC), HNRNPG, HNRNPA2B1, IGF2BPs, eIF3, and cytosol METTL3. Depending on the binding mechanism, they can be divided into three categories: direct binding, indirect binding, and m^6^A structural switch.^45^ The m^6^A switch refers to m^6^A-dependent RNA structural remodeling and RNA-protein interaction.^16^ Functionally, m^6^A modification is involved in RNA processing and maturation in the nucleus, affects RNA transport to the cytoplasm, and regulates RNA stability and translation in the cytoplasm. Moreover, some m^6^A “readers” coordinate these processes (Fig. 3).

#### RNA processing

Before RNAs mature, precursor mRNA (pre-mRNA), pre-miRNA and primary miRNA (pri-miRNA) experience alternative processing events, such as the splicing of RNA, the 5′-capping and 3′-polyadenylation of mRNAs, and the cleavage of miRNA by RNase III enzyme Drosha and Dicer successively. Dominissini et al.^43^ reported that *METTL3*-knockdown (KD) in HepG2 cells results in the differential expression of transcript isoforms with enriched m^6^A peaks, suggesting that m^6^A may affect splicing. Moreover, compared to METTL3 depletion, FTO depletion significantly affects m^6^A levels in the surrounding regions of constitutive and alternative splice sites.^60^ The underlying mechanism is that FTO-dependent m^6^A sites spatially overlap with the sites of exonic splicing enhancers (ESEs), which results in co-recruiting the splicing factors SRSF1/2. Bartosovic et al.^61^ also observed m^6^A-mediated exon-skipping events in *FTO*-knockout (KO) cells. Since then, multiple studies have confirmed the role and mechanism of m^6^A in RNA splicing and maturation.

The nuclear “writer” YTHDC1 reportedly interacts with five other splicing factors (SRSF1, SRSF3, SRSF7, SRSF9, and SRSF10). YTHDC1-SRSF3 tends to promote exon inclusion, while YTHDC1-SRSF10 promotes exon skipping.^62^ Interestingly, the nuclear AURKA interaction with HNRNPK and YTHDC1 inhibits *RBM4* exon inclusion by m^6^A-YTHDC1-SRSF3 and results in *RBM4* exon skipping in an m^6^A-YTHDC1-HNRNPK-dependent manner.^63^ In addition, YTHDC1 is associated with pre-mRNA 3′-end processing factors, including CPSF4, CPSF6, SRSF3, SRSF7, and FIP1L1, to inhibit alternative polyadenylation and alter the 3′-UTR length of the target transcript.^64,65^ Similarly, NKAP, acting as an m^6^A “reader”, combines with m^6^A and recruits splicing factor SFPQ for transcription termination site (TTS) splicing.^66^ Moreover, m^6^A also affects the processing of miRNAs. The microprocessor Drosha-DGCR8 complex plays a critical role in processing pri-miRNAs into pre-miRNAs, a first step in miRNA biogenesis. Tavazoie and colleagues^67^ observed that METTL3 depletion leads to a downregulation of overall mature miRNAs and a significant upregulation of pri-miRNAs. They also revealed the underlying mechanism that DGCR8 effectively recognizes and binds pri-miRNAs containing METTL3-labeled m^6^A markers, facilitating the processing of most pri-miRNAs into pre- and mature miRNAs. Furthermore, it is reported that m^6^A “readers”, such as HNRNPA2B1 and NKAP, play an important role in the METTL3-mediated miRNA maturation by recognizing and directly binding m^6^A sites (especially RGAC motif) to recruit the DGCR8.^17,68^ This mechanism does not appear to be unique to METTL3-mediated m^6^A, as the same phenomenon is observed in cells overexpressing METTL14 where METTL14 is proved to interact with DGCR8,^69^ or to benefit the interaction between DGCR8 with pri-miRNAs.^70^ Another mechanism is that m^6^A-mediated RNA structural changes regulate the RNA-structure-dependent accessibility (also known as m^6^A-switch) to some RBPs conducting RNA processing events, such as HNRNPC and HNRNPG. In this case, m^6^A results in the exposure of HNRNPC and HNRNPG preferential binding sites, which promotes splicing of nearby exons.^16,71^ Moreover, HNRNPG binds to the m^6^A-modified pre-mRNA, which prolongs the RNA polymerase II (RNAPII) occupancy near the exon-intron junction and is attributed to the recruitment of spliceosome components or splice factors.^72^

Although “readers” may be primary regulators of m^6^A-mediated alternative splicing, the role of “writers” and “erasers” cannot be ignored. METTL16 is identified as the methyltransferase of *MAT2A* and *U6* spliceosomal small nuclear RNA (snRNA). METTL16-mediated methylation of MAT2A hairpins (hp), especially its occupancy on hp1 in *MAT2A* 3'UTR, is crucial in inducing alternative splicing of *MAT2A*,^73^ while METTL16-mediated m^6^A modification of *U6* snRNA at A43 may be related to 5'-splicing site recognition on pre-mRNA.^51,74^ Tang et al.^75^ reported that about 41% of m^6^A sites overlap with exon skipping/inclusion or intron skipping/retention, enriched near the sites where abnormal splicing events occur (such as producing the aberrant shorter transcript isomers in spermatocytes). They found that ALKBH5-dependent m^6^A demethylation regulates the correct splicing of long 3′UTR transcripts. METTL3 typically mediates the m^6^A modification of an intron at the 5'-splicing site, which enhances its downstream splicing, suppresses upstream splicing, and may provide a basis for specifying other splicing outcomes.^76^ However, more evidence is needed to explore specific mechanisms.

#### RNA nuclear export

Mature nuclear mRNAs and most ncRNAs require transport into the cytoplasm to function. It was observed that nuclear mRNA output in ALKBH5-deficient cells is accelerated with cytoplasmic mRNA level significantly increased, which may be the result of the phosphorylation of SF2/ASF (coded by SFRS1) to switch from splicing factors to export adapter proteins in an m^6^A-dependent manner.^59^ This mechanism can also be applied to SRSF3. Roundtree and colleagues reported that YTHDC1-SRSF3 mediates the nuclear export of m^6^A-mRNA.^77^ They found that YTHDC1 facilitates the binding of the mRNA export receptor NXF1. These results explain why *YTHDC1*-KD accumulates m^6^A-mRNAs in the nuclear, while they deplete in the cytoplasm. Moreover, YTHDC1 promotes the nuclear export of m^6^A-circRNAs, such as *circNSUN2*^35^ and *circSPECC1*.^78^ In addition, the m^6^A “reader” FMRP, containing nuclear localization and export sequences, is reported to preferentially bind target mRNAs with m^6^A modification and interact with nuclear export protein CRM1 to promote nuclear export of target mRNAs.^79^

#### RNA stability

The stability of RNA protected by a 5′-cap structure and a 3′-poly(A) tail plays a critical role in controlling RNA metabolism. It is reported that m^6^A significantly shortens the half-life (2.5 h shorter on average) of RNAs and increases the decay rate (to 9 from 5.4 h on average) of mRNAs.^80^ m^6^A’s preference for the last exon of transcripts, especially the longer one, may be related to mRNA stability, as it facilitates the selection of 3′-poly(A) sites which play important roles in promoting translation and preventing mRNA degradation.^81^ Slobodin and colleagues^82^ revealed that both m^6^A and CCR4-NOT deadenylase complex regulate poly(A) tail length and mRNA stability. Moreover, m^6^A “readers” are reportedly involved in deacetylation-mediated mRNA decay. For instance, YTHDF2 recruits the CCR4-NOT complex through a direct interaction between its N terminus and the superfamily homology (SH) domain of CNOT1, a scaffold subunit of the CCR4-NOT complex, thus destabilizing m^6^A-containing RNAs by accelerating deadenylation.^83^ In contrast, IGF2BP1 inhibits deadenylation-mediated mRNA degradation by competitively binding to PABPC1, a poly(A)-binding protein (PABP), to prevent CCR4-NOT complex recruitment to PABPC1, thereby enhancing m^6^A-modified mRNA stability and expression.^84^ FMR1 reportedly interacts with the PABPs to polyadenylate/deadenylate m^6^A-mRNAs and thus affects their stability.^85^

YTHDC1 reportedly regulates nonsense-mediated mRNA decay (NMD) based on m^6^A modification near the initiation codon,^86^ and destabilizes the subsets of m^6^A-labeled chromosome-associated regulatory RNAs (carRNAs) via the nuclear exosome targeting (NEXT) complex,^87^ and the m^6^A-labeled polyadenylated RNAs (such as *MYC* mRNA) via the poly(A) tail exosome targeting (PAXT) complex.^88^ YTHDC2 is found to have RNA-induced ATPase activity and 3′→5′ RNA helicase activity, and it interacts with 5′→3′ exoribonuclease XRN1 to regulate RNA decay.^89^ Furthermore, m^6^A “readers” recognize and bind with m^6^A modified RNAs, which can locate protein-RNA complex to RNA decay sites, thereby controlling the metabolism of RNAs. For example, YTHDF2 plays a dominant role in m^6^A-mediated RNA stability. *YTHDF2*-KD results in an accumulation of untranslated mRNAs, with an increased m^6^A/A ratio of the total mRNA, and a prolonged lifetime of the target mRNA, confirming that YTHDF2 destabilizes the m^6^A-containing mRNA. Specifically, the C-terminal domain of YTHDF2 selectively binds to m^6^A-containing mRNAs, while the N-terminal domain helps its targeted mRNAs to localize from the translatable pool to mRNA decay sites (such as processing bodies) for sustained degradation.^18^ YTHDF2 mediates mRNA decay, by directly interacting m^6^A-mRNA and argonaute RISC catalytic component 2 (AGO2),^90^ or by indirectly recognizing m^6^A-circRNA.^91^ Moreover, YTHDF1/3 are also involved in YTHDF2-mediated RNA degradation.^90,92^ Depletion of YTHDF2 or any two YTHDFs or all three of them results in increased stability of m^6^A-mRNA, but they may be cell-specific.^93,94^ Recent studies have demonstrated that YTHDF1/3 attenuates the stability of target transcripts.^95–98^ These results suggest that YTHDFs play an important role in m^6^A-mediated RNA stability, but their biological function may vary from cell to cell, where they may cooperate, compensate or compete with each other. More evidence is needed to verify this assumption. Moreover, both FMRP and MSI2 act as m^6^A “readers” to protect their targeted m^6^A-mRNAs from YTHDF2-dependent degradation,^99,100^ but the mechanisms have not been clarified. In contrast to YTHDFs-mediated RNA decay, m^6^A modified target mRNAs of IGF2BPs appear to be more stable by recruiting the RNA stabilizers such as HuR, MATR3, PABPC1, and stress granule marker TIAR,^101–103^ or by inhibiting the miRNA-dependent decay.^104,105^ Other studies showed that some ncRNAs (such as lncRNAs and cirRNAs) are also involved in m^6^A-mediated mRNA stability by interaction with YTHDF2 or IGF2BPs,^106–111^ but the specific mechanism remains unclarified. In addition, m^6^A affects the binding of RNA stabilizers to target mRNAs (such as *ETS1*, *ZMYM1*, and *GATA3*) and ncRNAs (such as *DDIT4-AS1*), thus regulating transcript stability.^112–115^ For example, m^6^A repels the HuR binding to U-rich regions at 3′UTR and the G3BP1 interaction with GG(m^6^A)CU-containing targets.^116,117^ Thus, *MELLT3*-KD enhances the stability of both HuR and G3BP1 target RNAs.

#### mRNA translation

Translation events mainly include initiation (TI), elongation, and termination. Depending on the mechanisms of TI, there are cap-dependent and independent translations. The former is mediated by cap-binding complexes such as the eukaryotic initiation factor 4F (eIF4F) and CBP80/20 complexes.^118,119^ The internal ribosome entry site (IRES) trans-acting factors (ITAFs, such as G3BP1) mediates translation in a cap-independent manner, also known as IRES-dependent translation.^118^ m^6^A reportedly regulates mRNA translation through various mechanisms affecting initiation and elongation.

The classic mode of translation initiation in eukaryotic cells is that eIF4E, (a subunit of eIF4F complex) binds to the 5′-cap and recruits the 40S ribosomal subunit to form a 43S preinitiation complex with the help of eIF3.^118^ In the cytoplasm, METTL3 reportedly acts as an m^6^A “reader” to regulate the target mRNA translation (such as *EGFR* and *TAZ*) in CBP80/20-dependent and eIF4E-dependent manners by interaction with eIF3,^120^ and “reader” METTL3 facilitates cap-dependent translation by binding with eIF3H subunit to 3′UTR specific sites near the stop codon and leading to mRNA circularization.^121^ Similarly, cytoplasmic METTL16 exerts a methyltransferase activity-independent function in regulating translation. Mechanistically, the Mtase domain of METTL16 binds to the 5′-cap and interacts with eIF3A/B and 18S rRNA.^122^ Cytoplasmic METTL16 promotes eIF4E-dependent translation by interacting with eIF4E2 and repressing eIF4E2’s competition with eIF4E for cap binding.^123^ YTHDF1-mediated translation relies on the interaction with eIFs (such as eIF3) and the eIF4G-dependent loop formation.^124^ YTHDF1 promotes cap-independent translation of mRNA with m^6^A in CDS by binding the elongation factor eEF2.^125^ Moreover, RNA helicase-containing m^6^A “reader” YTHDC2 is required for elongation of mRNA with m^6^A in CDS by resolving the mRNA structural hurdle and facilitating ribosome removement.^126^ It has been shown that YTHDF3 also participates in cap-independent translation by interacting with the 40S and 60S ribosomal proteins rather than eIFs.^92,127,128^ This mechanism also applies to the translation of circRNAs in response to stress.^129^ More importantly, m^6^A provides a new mechanism for translation in a cap-independent manner (also known as m^6^A specific translation), which is distinct from cap- or IRES-dependent translation. Under this condition, eIF3 directly binds m^6^A for cap-independent ribosome recruitment.^118^ Meyer and colleagues^130^ found that the 5′-cap and eIF4E are not indispensable for the translation of 5′UTR m^6^A-mRNA (such as *Hsp70* mRNA in mouse embryonic fibroblasts), which directly recruits the 48S complex (composed of eIF1, 1A, 2, 3, and 40S subunit) and selectively initiates translation from the first AUG.

In addition, METTL5 and ZCCHC4 mediate methylation of 18S rRNA and 28S rRNA, which affects the 40S ribosome structure, 80S activity, and occupancy of specific codons, thereby regulating global translation.^131–134^ If m^6^A is added to the mRNA codon, it prolongs cognate tRNA decoding, which depends on the position and context of m^6^A in the codon.^135^

### N^1^-methyladenosine (m^1^A)

m^1^A is when methyl is added to the N^1^ position of adenosine. Half a century ago, m^1^A was identified primarily in tRNAs and rRNAs of various types of fungi and bacteria. In 2016, Dominissini and colleagues^23^ explored m^1^A in mammalian mRNAs using MeRIP-seq based on the m^1^A antibody. Their results showed that m^1^A is mainly distributed in the 5′UTR and CDS regions, and methylated transcripts contain more alternative TISs, suggesting that m^1^A may be involved in the initiation of translation. However, Grozhik et al.^136^ point out that m^1^A is not a prevalent high stoichiometric modification in mRNAs, and there may be false positives in m^1^A antibody-based on mapping methods. They found that m^1^A is difficult to detect at the transcription-start nucleotide, and that the m^1^A antibody specifically interacts with the m^7^G-cap of mRNA in an m^1^A-independent manner, which may explain why m^1^A peaks are displayed at the 5′UTR.

**Fig. 4:**
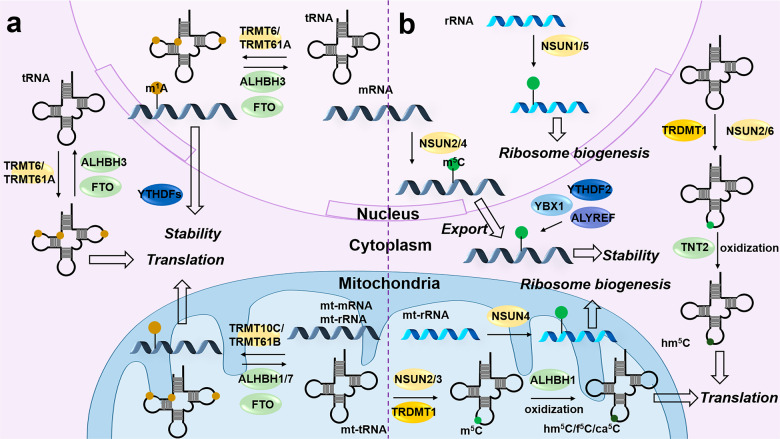
Dynamic m^1^A and m^5^C modification on RNAs. **(a)** TRMT6/TRMT61A are m^1^A methylases of mRNAs and tRNAs in the nucleus. TRMT10C/TRMT61B catalyze m^1^A on mitochondrial (mt-) tRNA, rRNA and mRNA (such as *ND5* mRNA). ALKBH1/3/7 and FTO regulate their demethylation. Functionally, m^1^A mediates RNA stability and translation via various mechanisms. YTHDFs may function as m^1^A “readers”. **(b)** NSUN1-6 are “writers” for RNA m^5^C modification to regulate the nuclear export, metabolism and functions of RNA. ALYREF, YBX1, and YTHDF2 reportedly act as m^5^C “readers”. ALKBH1 and TNT2 are responsible for the oxidizing, rather than the removing m^5^C to hm^5^C, f^5^C, and ca^5^C

The m^1^A “writer” TRMT61B is the first identified methyltransferase modifying both tRNAs and rRNAs in humans,^137,138^ while TRMT10C is responsible for the m^1^A methylation of tRNAs (Fig. 4a).^139^ Subsequent studies reported that both TRMT61B and TRMT10C regulate m^1^A levels in mitochondrial mRNAs.^140,141^ It should be noted that m^1^A in mitochondrial transcripts is enriched mainly in the CDS region and the third position of a codon, affecting translation in mitochondria. Moreover, these studies demonstrated that the TRMT6/TRMT61A-dependent m^1^A has a high sequence preference within the cytosol, predominantly distributed in a GUUCRA motif with a strong hairpin structure, identified as the tRNA T-loop. Like m^6^A, m^1^A modification is a reversible process, which is removed by “erasers” such as ALKBH3.^142^ ALKBH3-mediated demethylation of *CSF-1* mRNA significantly improves the stability of *CSF-1* in breast and ovarian cancer cells,^143^ while in human retinal pigmented epithelial 1 (RPE-1) cells, ALKBH3 deletion inhibits the *Aurora A* mRNA stability as well as its translation.^144^ Also, ALKBH3-mediated demethylation of tRNA is crucial for translation initiation and promotes the generation of tRNA-derived small RNAs (tDRs).^145^ These results suggest that ALKBH3 can regulate transcriptional degradation and translation in an m^1^A-dependent manner by various mechanisms. Another “eraser” ALKBH1 is identified as a demethylase of tRNAs. The ALKBH1-mediated demethylation of tRNAs at position 58 affects their stability and translation, including translation initiation by influencing the assembly of the 80S monomers, and translation elongation by eEF1A promoting the enrichment of tRNAs in the active translation pool.^146^ ALKBH7 also has demethylase activity and its substrate is mitochondrial pre-tRNA.^147^ FTO is also a demethylase of nuclear and cytoplasmic m^1^A-tRNAs and is selective for stem-loop structures, which promote protein translation efficiency.^148^ Furthermore, serum starvation and H_2_O_2_ stimuli result in specific m^1^A peaks, suggesting that different stress conditions may regulate m^1^A in mRNAs.^142^ Similarly, glucose deprivation leads to reduced m^1^A-tRNA levels in cells, thereby inhibiting translation, which can be reversed by *ALKBH1*-KD.^146^

### 5-methylcytosine (m^5^C)

m^5^C has been once thought to be one of the DNA markers. With the development and application of detection technologies (recently reviewed in detail by Guo et al.^149^), m^5^C is widely found in eukaryotic RNAs as well, especially tRNAs and rRNAs (Fig. 4). In mammalian RNAs, m^5^C “writers” mainly include NOL1/NOP2/SUN (NSUN) family members and tRNA aspartic acid methyltransferase 1 (TRDMT1, also known as DNMT2). NSUN2 is mainly responsible for mRNAs (especially in CG-rich and CDS regions), tRNAs and microRNAs.^20,29,150,151^ NSUN3, NSUN6 and TRDMT1 are the main methyltransferases of tRNAs.^150,152–154^ NSUN1 and NSUN5 target rRNAs.^155,156^ NSUN4 catalyzes mRNA and mitochondrial rRNA.^157,158^ The Aly/REF export factor (ALYREF, an mRNA transport adapter), YTHDF2, and Y-box binding protein 1 (YBX1) are identified as m^5^C “readers”.^19,20,159^ The controversy about the m^5^C “erasers” still exists. It is unclear whether m^5^C modification is a reversible process like m^6^A or m^1^A, but some studies found that m^5^C can be oxidized, rather than demethylated, to 5-hydroxymethylcytosine (hm^5^C), 5-formylcytosine (f^5^C) and 5-carboxylcytosine (ca^5^C) by ALKBH1 and ten-eleven-translocator (TET) enzymes.^9,160–162^ Functionally, m^5^C, together with its oxidized derivatives, regulates RNA nuclear export, stability, processing, and mRNA translation, thus participating in the organ development and function, as well as the occurrence and progression of diseases such as cancer, mitochondrial disease, infection, and colitis.^20,152,159,163–165^

### N^7^-methylguanosine (m^7^G)

**Fig. 5:**
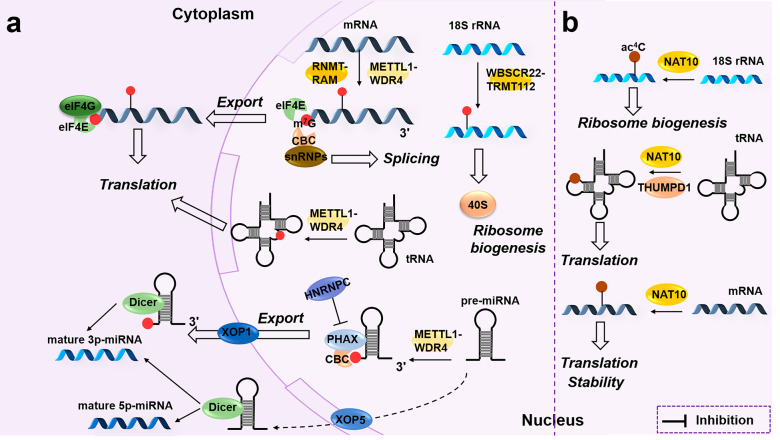
m^7^G and ac^4^C modification on RNAs. **a** In the nucleus, METTL1-WDR4 are m^7^G “readers” of mRNA, pre-miRNA and tRNA. RNMT-RAM mainly targets mRNA, while WBSCR22-TRMT112 is responsible for 18S rRNA. Functionally, m^7^G facilitates RNA nuclear export, translation, 40S ribosome biogenesis, and selective miRNA maturation. **b** In the nucleus, NAT10-THUMPD1 catalyzes ac^4^C mainly on rRNA, tRNA and mRNA, which is related to ribosome biogenesis, translation and mRNA stability, respectively

As early as fifty years ago, m^7^G was identified as a post-translational modification widely seen in the 5′-cap structure of mammalian mRNA, typically connected with a triphosphate and 2′-O-methyl (N_m_) modifications.^166,167^ m^7^G is also found in ncRNAs such as miRNA, tRNA, and rRNA. In mammals, m^7^G “writers” mainly include METTL1, WD repeat domain 4 (WDR4), Williams-Beuren syndrome chromosome region 22 protein (WBSCR22, coded by *BUD23* gene), TRMT112, RNA guanine-7 methyltransferase (RNMT) and RNMT-activating miniprotein (RAM). RNMT-RAM primarily targets mRNA;^168^ WBSCR22-TRMT112 adds m^7^G to 18S rRNA;^169^ and METTL1-WDR4 are responsible for mRNA, tRNA, and miRNA.^170–172^ Functionally, m^7^G is thought to regulate the export and translation of mRNA and the maturation of 18S rRNA (Fig. 5a).

The m7G cap structure primarily determines mRNA’s stability, splicing, and translation. It is demonstrated that m^7^G protects mRNA from 5′→3′ exonuclease degradation and promotes splicing.^173–175^ Pabis and colleagues^176^ found that m^7^G-mediated splicing is due to the role of CBC in recruiting snRNPs and spliceosome assembly. As discussed above, cap-dependent translation initiation is mediated by the cap-binding protein eIF4E. The eIF4E affinity to the m^7^G cap was enhanced by the interaction of eIF4G peptides with the cap.^177^ The middle domain of the AGO2 protein is similar to the cap-binding domain of eIF4E and can bind specifically to the m^7^G cap, which impedes the recruitment of eIF4E and thus inhibits translation initiation.^178^ The endogenous let-7 microribonucleoproteins (miRNPs) may play a similar role in m^7^G cap-dependent translation.^179^ Moreover, METTL1-WDR4 can add m^7^G to the internal of target mRNAs, which facilitates translation.^172^ In mice, m^7^G is enriched in tRNAs with the RAGGU motif, affecting translation.^180^ METTL1-mediated m^7^G-tRNA promotes the use of m^7^G codons in mRNA translation.^171,181^

Moreover, the m^7^G cap also affects RNA nuclear export and miRNA maturation. In the nucleus, eIF4E regulates RNA export. For example, eIF4E is physically associated with cyclin D1 mRNA and promotes its transportation.^182^ CBC-PHAX binds to m^7^G-capped pre-miRNAs and facilitates their transportation via XPO1 rather than via the canonical XPO5 pathway.^183,184^ In the cytoplasm, only mature 3p-miRNA is produced along with the extended 5p-miRNA.^183^ In addition, HNRNPC may inhibit PHAX activity through interactions with CBC.^185^

### N^4^-acetylcytosine (ac^4^C)

N^4^-acetylcytosine is thought to be the only acetylation in post-transcriptional modification. In the past, ac^4^C has been explored mainly in bacterial and fungal tRNA and rRNA. The discovery of N-acetyltransferase 10 (NAT10) promotes the exploration of RNA ac^4^C modification in mammals (Fig. 5b). In HeLa cells, NAT10 reportedly mediates ac^4^C_1842_ in 18S rRNA in an ATP-dependent manner. It regulates ribosome biogenesis, but the exact mechanism remains to be investigated further.^186^ A recent study reported that SNORD13 assists ac^4^C_1842_ in 18S rRNA, but the mechanism has not been clarified.^187^ In human colon cancer cells, NAT10 depletion significantly reduces tRNA acetylation.^188^ Moreover, the NAT10-THUMPD1 complex is responsible for acetylating human tRNA, whereas THUMPD1 primarily acts as a tRNA adapter. With the development of ac^4^C detection technologies, Arango and colleagues^189^ identified ac^4^C-modified mRNAs in HeLa cells in 2018. Their results show that ac^4^C significantly enhances mRNA stability through uncoupled exonuclease resistance, particularly in the CDS. The translation of ac^4^C-mRNA is significantly enhanced, possibly mediated by the enhanced mRNA stability rather than the 18S rRNA and tRNA^ser/leu^ acetylation. Moreover, if ac^4^C is present at the wobble site of the codon, it is conducive to cognate tRNA recognition, significantly improving the decoding efficiency. Schwartz et al.^190^ comprehensively mapped the ac^4^C modification in eukaryotic RNA subsequently. They found that the ac^4^C site in mRNA, rRNA, and tRNA, induced by NAT10-THUMPD1 overexpression, almost occurs at the CCG motif and that ac^4^C is absent or maintained at relatively low levels in endogenous eukaryotic mRNA. A recent study pointed out that acetylation in 5′UTR promotes the translation initiation in upstream sequences.^191^ These results suggested that ac^4^C in different regions of mRNA may affect translation through different mechanisms. NAT10 also mediates the acetylation of lncRNA CTC-490G23.2 in esophageal squamous cell carcinoma.^192^

The role of mammalian ac^4^C-modified RNA in the occurrence and development of diseases has been gradually revealed. NAT10-mediated ac^4^C positively regulates runt-related transcription factor 2 (RUNX2) mRNA stability and protein expression, thus promoting the differentiation of bone marrow-derived mesenchymal stem cells (BMSCs).^193^ Similarly, NAT10 is dysregulated in various cancers, which mediates ac^4^C modification of target mRNA (such as *BCL9L, SOX4, AKT1, FSP1* and *KIF23*), and promotes cancer progression.^194–198^ In terms of therapeutic applications, ac^4^C is expected to be used in nucleic acid therapeutics, as Nance et al.^199^ found that ac^4^C significantly inhibits synthetic mRNA-mediated inflammation, which may be beneficial for repeated drug administration.

### Uridylation

Uridylation is a post-transcriptional modification mainly found in the poly(A) tail, especially the short one (5–25 nt), also known as the U-tail.^200,201^ This poly(A)-tail length-dependent uridylation may also be associated with PABPs, because PABPC1 preferentially binds RNAs with long poly(A) tail and against terminal uridylyl transferases (TUTs). There are exceptions that the 3′-uridylation modification also exits in histone mRNAs without poly(A) tails.^202,203^ Uridylation is added by TUTs and terminal nucleotidyl transferases (TENTs). Currently reported mammalian uridylation “writers” include TUT2, TUT4 (ZCCHC11), TUT7 (ZCCHC6), TENT2, TENT4B, and TENT5C. A recent study indicated that TUT4, rather than TUT7, is primarily responsible for the uridylation of mature miRNAs and that TENT2 selectively modifies mature miRNAs with little effect on their abundance.^204^

**Fig. 6:**
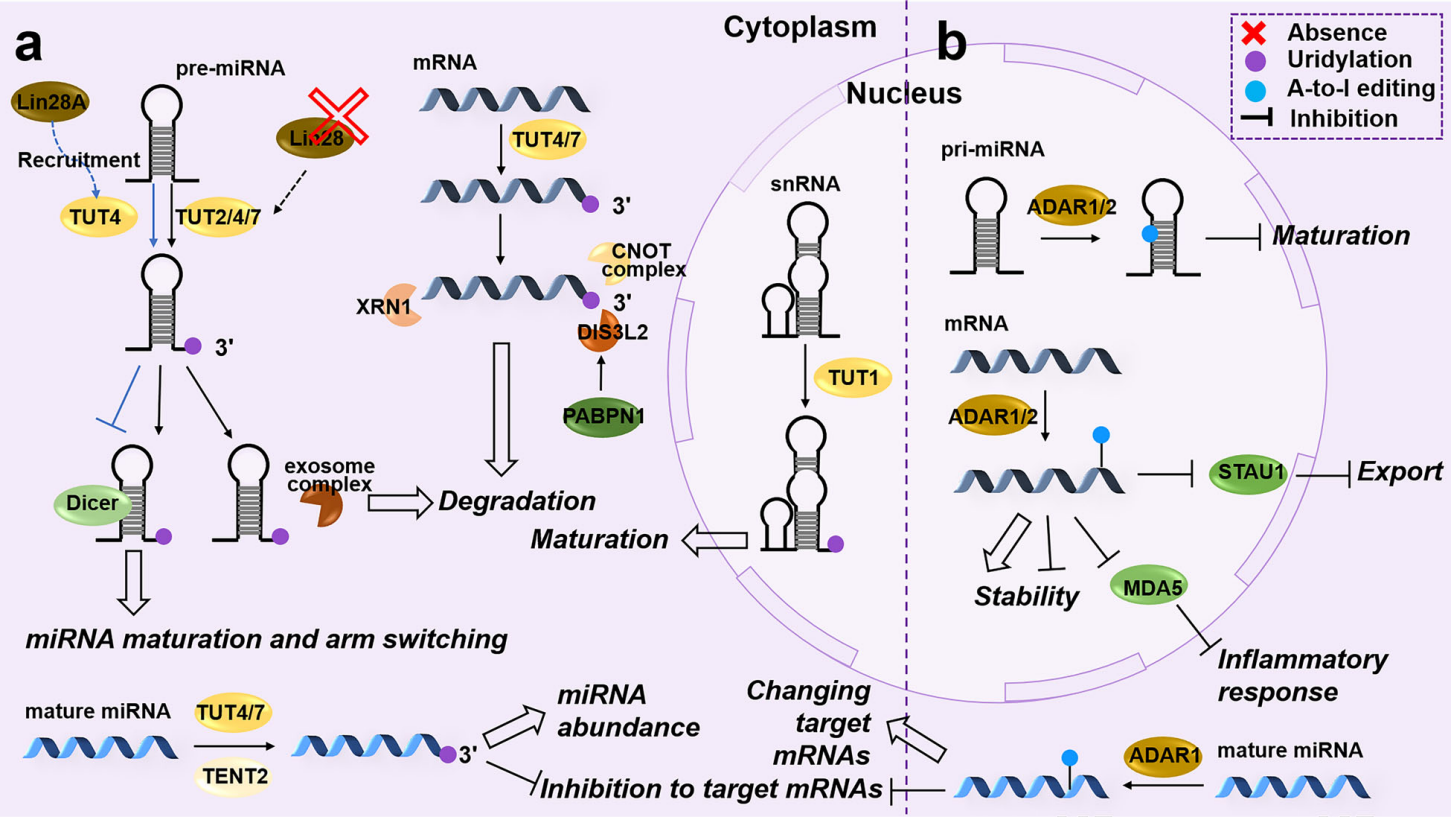
Uridylation and A-to-I editing on RNAs. **a** In the cytoplasm, TUT2/4/7 catalyzes the 3′-uridylation of precursor miRNA (pre-miRNA), mature miRNA, and mRNA, whereas TUT1 is a uridylation “writer” of snRNA in the nucleus. TENT2 is responsible for the mature miRNA. Uridylation regulates the maturation of miRNA and snRNA, the functions of miRNA, and RNA degradation. **b** ADAR1/2 plays as A-to-I editing “writers” of primary miRNA (pri-miRNA), mature miRNA, and mRNA, which affects the maturation and function of miRNA and the stability and export of mRNA

Uridylation is primarily present in mRNA and miRNA to influence their metabolism (Fig. 6a). Firstly, the role of deadenylases (e.g., CCR4-NOT complex) and PABPs-mediated deadenylation in RNA stability is described above (see Section “RNA stability”). Similarly, uridylation and deadenylation regulators mediate RNA metabolism through antagonistic or synergistic effects. CCR4 promotes the dissociation of PABPC1 from the poly(A) tail, which is conducive to mRNA decay mediated by TUTs or decay factors.^205^ In addition, miR-1, which acts as a deadenylation inducer, also affects uridylation and mRNA decay.^201^ TUT4/7 depletion significantly prolongs the half-life of miR-1 target mRNAs. The final step in mRNA degradation is handled by 5′ → 3′ (e.g., exoribonuclease XRN1) or 3′ → 5′ (e.g., exosomes and exoribonuclease DIS3L2) decay factors. XRN1 contributes to the degradation of mRNA with U-tail added by TUT4/TUT7.^201^ DIS3L2 preferentially degrades uridylated RNA.^206^ TUT-DIS3L2 surveillance (TDS) pathway plays a crucial role in quality control and degradation of cytoplasmic RNAs in the mammal.^207^ PABPN1 facilitates the 3′→5′ decay of uridylated mRNA by recruiting DIS3L2.^208^ Uridylated miRNAs are significantly enriched in exosomes and degraded.^209,210^ Exosomes promote the uridylation of AGO-bound pre-miRNAs by cooperating with TUT7/4 to recognize and degrade pre-miRNAs without function for quality control. Furthermore, some RBPs, such as HuR and zinc-finger proteins, interact with adenylate-uridylate-rich elements (AREs) and regulate RNA decay.^211–214^ In addition, TUT7 uridylates *Zc3h12a* mRNA (encoding ribonuclease Regnase-1) and indirectly regulates the stability of a subset of cytokines, including IL-6.^215^

Uridylation is also involved in miRNA biogenesis. In the cytoplasm, ribonuclease (RNase) III Dicer cleaves pre-miRNA formed by the Drosha-DGCR8 complex. Heo et al.^216^ found that Lin28 recruits TUT4 specifically to bind to the GGAG motif and uridylates cytoplasmic pre-let-7, which blocks Dicer processing and significantly reduces mature let-7. This mechanism also applies to miR-1.^217^ Interestingly, they later reported that in the absence of Lin28, such as in HeLa cells, TUT7/4/2 mediated 3′-strand (3p) mono-uridylation in most pre-let-7 and pre-miR-105 is essential for Dicer processing.^218^ Also, the uridylation frequency in 3p.1 has a negative correlation with the 5p/3p.1 ratio, because the 3p uridylation alters the Dicer cleavage site selection of pre-miR-324, leading to arm switching of miR-324.^219^ The uridylation of mature miRNAs influences their biological function. For example, uridylated miR-26a and miR-26b repress their inhibition of target genes such as *IL-6* mRNA.^37,220^

### Adenosine-to-Inosine (A-to-I) RNA Editing

Adenosine-to-inosine (A-to-I) RNA editing refers to the conversion of adenosine to inosine in RNA by adenosine deaminases acting on RNA (ADARs), which is widely found in double-stranded RNAs (dsRNAs) with Alu and LINE (long interspersed nuclear element) elements.^221,222^ RNA editing leads to changes in RNA secondary structure, amino acid sequence, alternative splicing, miRNA-target regulation, and gene expression, which can affect cell phenotypes and lead to diseases.^223–227^ The editing alters the RNA secondary structure at the Dicer cleavage site and inhibits miRNA biogenesis.^228^ In cardiomyocytes (CMs), edited miR-34a undoes its inhibition to target mRNAs.^229^ Various RBPs, such as HNRNPC, Drosha, and HuR, prefer or repel edited sites, connecting RNA editing events to RBP-mediated biological processes.^230–232^ Specifically, for example, edited *CTSS* mRNA benefits HuR recruitment with improved stability,^231^ while ADAR1 occupancy on repetitive Alu elements antagonizes their interaction with STAU1 and STAU1-mediated RNA export.^233^ This section mainly focuses on advances in the last decade to avoid excessive duplication, since Kazuko Nishikura detailed A-to-I editing and ADARs in mammals (see ref. ^221^).

ADAR1 and ADAR2 (ADARB1) have enzyme activity for A-to-I RNA editing (Fig. 6b). They are not mutually compensatory in vital functions nor have unique substrate preferences.^234^ ADAR1 acts as an oncogene in cancer, while ADAR2 has an inhibitory effect.^235,236^ They also play a similar role in circRNA regulation. ADAR1 inhibits or promotes circRNAs equally, but ADAR2 mainly acts as an inhibitor.^232^ Moreover, ADAR1 is dominant in editing. In human umbilical arterial fibroblasts (HUAFs), ADAR1 almost participates in the editing of all pri-microRNAs and mature microRNAs, whereas ADAR2 is only responsible for pri-miR-376a1, pri-miR-376a2, and miR-376a+b.^237^ ADAR2 inhibits the editing of 66 ADAR1 targets in the heart.^238^ There are two subtypes of ADAR1, p110 and p150, mainly distributed in the nucleus and the cytoplasm, respectively. In the nucleus, ADAR1p110 edits A-C mismatched base pairs of telomeric RNA: DNA hybrids, which facilitates RNase H2 processing of the telomere R-loop and promotes genomic stability.^239^ ADAR2 has a similar effect.^240^ Song et al.^241^ reported that ADAR2-mediated editing of *COPA* pre-mRNA results in an isoleucine-to-valine substitution at residue 164 in hepatocellular carcinoma, which produces a less stable protein and switches *COPA* from an oncogenic gene to a suppressor by deactivating PI3K/AKT/mTOR signaling. RNA editing alters the subcellular localization of the *AZIN1* protein from the cytoplasm to the nucleus, with differentially interacting proteins,^224,242^ and the target of the edited miRNAs will also be changed.^237,243^

An overall decrease in edited dsRNA levels may result in a higher risk of diseases, such as inflammatory bowel disease (IBD), coronary artery disease (CAD), diabetes, and Parkinson’s disease (PD).^244^ ADAR1, for example, is essential in regulating the inflammatory response. ADAR1-mediated RNA editing is indispensable for inhibiting interferon (IFN) response to maintain homeostasis. To a certain degree, it may contribute to the restraint on innate RNA sensor MDA5 (encoded by *IFIH1* gene) in recognition of unedited dsRNAs, as well as in the activation of PKR, ZBP1 and subsequent signaling.^222,245–247^

### Pseudouridine (Ψ)

Pseudouridylation is considered the most abundant post-transcriptional modification in RNA and it is the isomerization product of a uridine by pseudouridine synthases (PUSs), which are different from the above-mentioned “writers”.^248^ Pseudouridylation promotes RNA stability. It is a hardly reversible procedure, because the C-C bond in Ψ is more stable than the C-N glycoside bond in uridine.^249,250^ Ψ can be formed in an RNA-independent or RNA-dependent manner. The RNA-independent mechanism means that PUS specifically recognizes and directly binds to the specific sequence or structure of RNA,^251^ whereas the RNA-dependent manner relies on the box H/ACA snoRNA complexed with proteins (known as snoRNPs), including PUSs (such as Dyskerin).^252–254^ U23 and U71 snoRNA target 18S rRNA via 5′- or 3′-hairpin elements and mediate the pseudouridylation of uridine residues at positions 97 and 410, respectively.^255^ Furthermore, Schwartz et al.^256^ found that in HEK293T cells and fibroblasts, the Ψ sites of mRNA are mainly enriched in GUUC and UGUAG motifs, which are consistent with the sites modified by Pus4 in yeast and Pus7 in other mammals, respectively. Moreover, some sites are modified by Dyskerin and are complementary to snoRNAs. A recent study, based on bisulfite-induced deletion sequencing (BID-seq), showed that the mRNA Ψ sites mainly distribute in CDS and 3′UTR.^257^ Environmental stimuli, such as serum starvation, significantly modulate mRNA pseudouridylation in HeLa cells, indicating Ψ may be involved in cellular growth state regulation.^26^

**Fig. 7:**
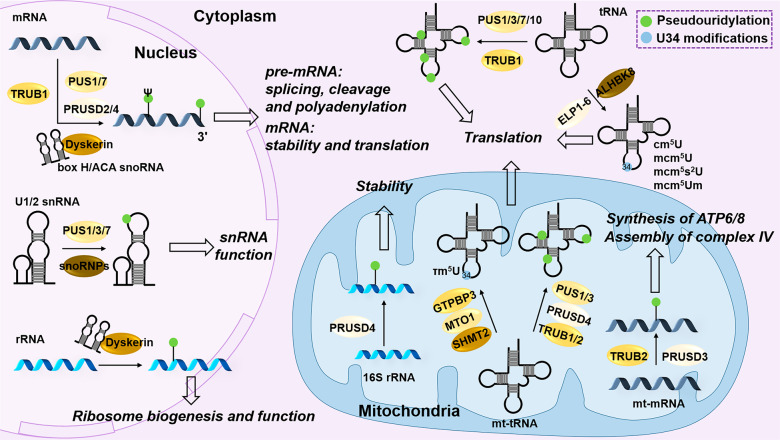
Pseudouridylation on RNAs and U34 modification on tRNA. The mammal pseudouridylation “writers” of RNA mainly include PUS1/3/7/10, PRUSD2-4, TRUB1/2, and Dyskerin. Dyskerin catalyzes pseudouridylation in a H/ACA snoRNA-dependent manner. Functionally, pseudouridylation is responsible for RNA processing, stability and functions. For tRNAs, U34 modifications, such as cm^5^U, τm^5^U, mcm^5^U, mcm^5^s^2^U and mcm^5^Um, play important roles in translation. ELP1-6 catalyzes cm^5^U methylated by ALKBH8 to produce mcm^5^U and its derivatives. GTPBP3 and MTO1 catalyze the τm^5^U of substrate mt-tRNAs, in which SHMT2 provides the raw material for methyl synthesis

In mammalian, some PUSs are reported with biological function, including PUS1, PUS3, PUS7, PUS10, TRUB family (Pus4 homologs) and Dyskerin (a Cbf5p orthologue and coded by *DKC1*) (Fig. 7). The catalytic substrates of human PUS1 include tRNAs and mRNAs with structure specificity.^251,258^ Human PUS3 catalyzes tRNAs, usually mediating Ψ38 and Ψ39.^259,260^ PUS7 mainly targets the UGUAR motif in mRNA and ncRNA, especially snoRNA.^5,261^ Human PUS7 mediating Ψ-tRNA also relies on structural specificity, in which the T-arm and the anticodon arm are indispensable.^262^ PUS10 regulates Ψ54 and Ψ55 of tRNAs in the cytoplasm.^263,264^ Similarly, TRUB1, localized in mitochondria, is also responsible for forming Ψ55 of various tRNAs.^265^ *TRUB1*-KO causes conformation changes in these mt-tRNAs, making them more sensitive to nuclease and impair mitochondrial translation. Dyskerin mediates the pseudouridylation of rRNA and mRNA in an H/ACA snoRNA-dependent manner.^266–268^ Antonicka et al.^269^ identified three mitochondrial PUSs: TRUB2, RPUSD3, and RPUSD4. In human 143B cells, RPUSD4 mainly affects the pseudouridylation and stability of 16S rRNA, as well as the mitochondrial assembly, while TRUB2 and RPUSD3 mainly mediate the pseudouridylation of mRNA, thus affecting the synthesis of ATP6 and ATP8 subunits and the assembly of complex IV, respectively. Although PUS1, PUS7, RPUSD2, and TRUB2 can all mediate the Ψ-mRNA in humans, most modified sites are regulated by TRUB1 predominantly in nuclear,^270,271^ which Ψ-mRNAs.^257^ Also, it is important to note that the substrates of PUSs may be cell- and tissue- specific. Recently, methionine aminoacyl tRNA^Met^ synthetase (MetRS) reportedly functions as a pseudouridylation “writer” in yeast, which recognizes and binds Ψ by PUS6 in tRNA and mRNA,^272^ suggesting that there may exist other Ψ “writers” in mammals waiting to be explored.

A crucial biological function of Ψ is to regulate translation. It is reported that tRNA pseudouridylation regulates translation. First, PUS7 deletion significantly reduces specific tRNA-derived fragment (tRF) subgroups. For example, 5′-tRFs derive from tRNA containing a 5′-terminal oligoguanine (TOG), which are considered protein synthesis inhibitors by impairing eIF4E-mediated cap-dependent translation initiation.^5,273^ Cui et al.^261^ observed that PUS7-mediated tRNA Ψ inhibits codon-specific translation in glioblastoma stem cells (GSCs). In GSCs, *PUS7*-KO does not affect the overall translation efficacy, but significantly improves the translation of its target tRNA-Arg-CCG. Moreover, rRNA’s pseudouridylation also affects mRNA translation. The reduction of Ψ-rRNA in *DKC1*-deficient cells results in translation defects in specific mRNAs, such as p27, XIAP and Bcl-xL, in an IRES-dependent manner.^274^ The potential mechanism is that Ψ-rRNA directly affects the interaction between these mRNAs and the 40S subunit, thus regulating the assembly of 48S.^275^ In addition, the H/ACA snoRNA *SNORA24*-guided modification of 18S rRNA affects the ribosome’s efficiency of tRNA selection and the accuracy of translation in a codon-specific manner.^276^ Furthermore, the position of Ψ in mRNAs also determines different translation regulation mechanisms. In HEK293T cells, Ψ in mRNA codon increases the decoding rate of near-cognate tRNA and promotes protein synthesis.^277^ As an alternative translation regulation mechanism, Ψ in the termination codon significantly suppresses translation termination.^257,278^ Also, Ψ is beneficial to translation by inhibiting the activation of RNA-dependent protein kinase R (PKR), thus blocking the phosphorylation of eIF2A and promoting translation initiation.^279^ Furthermore, 2-thiouridine (s^2^U) and m^5^C have a similar effect, while 5-methyluridine (m^5^U) and m^6^A may play opposite role. Studies revealed that Ψ modification of snRNAs is important for their functions. For example, human Ψ-U1 helps a noncanonical uridine-pseudouridine interaction in the 5′-splice site and facilitates recognition.^280^ Ψ in U2 snRNA participates in spliceosome assembly^280^ and contributes to the binding and activity of the RNA-dependent ATPase Prp5,^281^ thus facilitating pre-mRNA splicing. Moreover, Martinez and colleagues^282^ observed that PUS1, PUS7 and RPUSD4-mediated Ψ enriches in the alternative splicing regions and near splice sites of pre-mRNA, which directly affects the splicing efficiency, which may be attributed to the Ψ sites overlapping with RBP sites (including splicing factors such as U2AF2) and the promoted RBP binding. Also, Ψ in 3′UTR probably regulates the cleavage and the polyadenylation of pre-mRNAs.

### Modifications of U34 on tRNA

Anticodons are located at positions 34 to 36 in tRNAs, and identify codons in mRNA strictly following Watson-Crick pairing. There is one exception, however, where the anticodon at position 34 does not always obey the rule, which is known as wobble pairing. Modifications at position 34 are crucial in regulating wobble pairing, affecting protein translation and participation in some essential parts of life activities. Moreover, U34 enzymes-mediated tRNA modification is required to decode -AA codon and regulate codon-specific translation.^6^ The loss of U34 enzymes results in translation elongation defection, misfolded protein accumulation, and unfolded protein responses (UPRs).^283,284^ Tsutomu Suzuki^31^ mapped the post-transcriptional modifications of human tRNA and illustrated how aberrant tRNA modifications can lead to diseases in detail. Based on this and the modifications of tRNA mentioned above, this section will mainly focus on modifications, such as 5-methoxycarbonylmethyluridine (mcm^5^U) and its derivatives, 5-taurinomethyluridine (τm^5^U), and 5-carboxyaminomethyluridine (cmnm^5^U), at U34 on human tRNAs (Fig. 7).

Elongator complex (ELP1-6) catalyzes 5-carbamoylmethyluridine (cm^5^U) in the cytoplasm. Then, ALKBH8 is responsible for methylation, which corporately leads to the formation of mcm^5^U and its derivatives, including 5-methoxycarbonylmethyl-2-thiouridine (mcm^5^s^2^U) and 5-methoxycarbonylmethyl-2′-O-methyluridine (mcm^5^Um). The deficiency of ALKBH8 results in stop codon recoding and selenoprotein synthesis by impairing tRNA^Sec(UGA)^.^285^ In breast cancer, ELP3-regulated mcm^5^s^2^U-tRNA modification of the Elongator subunit is required for IRES-dependent expression of genes associated with tumor cell invasion and metastasis.^286^ Moreover, ELP5, without enzymatic activity, is the critical subunit connecting ELP3 and ELP4. ELP5 depletion disrupts the integrity and stability of the Elongator complex, and ELP5-mediated the U34 tRNA modification cascade.^287^

In mitochondria, the point mutations cause mt-tRNA^Leu(UUR)^ lacking taurine modifications, including τm^5^U and 5-taurinomethyl-2-thiouridine (m^5^s^2^U), which significantly reduces UUG decoding with respiratory chain complex I deficiency by impairing the formation of codon-anticodon base pairs on the ribosomal A-site.^288^ GTPBP3 and MTO1 catalyze the τm^5^U of substrate mt-tRNAs. The human MTU1 (TRMU) is responsible for s^2^U34 on mt-tRNAs. Moreover, the τm^5^s^2^U is formed when both τm^5^U and s^2^U modifications exist. Mutations in GTPBP3 cause a decrease steady-state levels of respiratory complexes I and IV, as well as a defection in mitochondrial translation.^289,290^ The loss of mitochondrial folate enzyme serine hydroxymethyltransferase 2 (SHMT2) also affects the production of τm^5^U, and reduces the abundance of complex I, IV and V subunits because SHMT2-produced 5, 10-methylenetetrahydrofolate acid provides the raw material for methyl synthesis.^30^ However, MTU1 does not affect mitochondrial translation in human fibroblasts.^291^ Interestingly, mt-tRNA^Met^ at U34 modified with 5-formylcytidine (f^5^C) can decode isoleucine codons AUU and AUC to methionine.^292,293^

## Roles of RNA modification in cardiovascular diseases

**Fig. 8:**
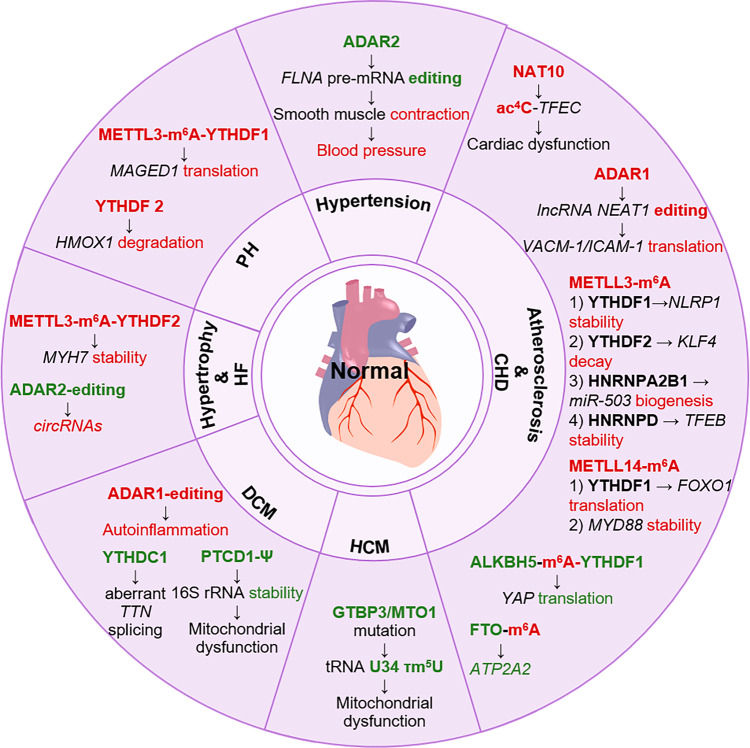
RNA modifications in cardiovascular diseases. RNA modifications, especially m^6^A and A-to-I RNA editing, play crucial roles in cardiovascular diseases such as hypertension, atherosclerosis, CHD, HCM, and DCM. The total m^6^A levels in CHD significantly increase with the upregulation of m^6^A “writers” and downregulation of “erasers”. ADAR1 is increased in CVD, whereas ADAR2 is decreased. Functionally, RNA modifications participate in vascular inflammation, angiogenesis, cell death and proliferation, and cardiac functions. The red font represents upregulation/promotion, while the green font represents downregulation/inhibition. CVD cardiovascular disease, CHD coronary heart disease, HCM hypertrophic cardiomyopathy, DCM dilated cardiomyopathy, HF heart failure, PH pulmonary hypertension

Cardiovascular disease (CVD) refers to a series of diseases affecting the heart and blood vessels, which is the leading cause of global death and contributes to a large global disease burden. Prevalence and mortality of CVD increased significantly in patients over 40.^294,295^ Hypertension and atherosclerosis belong to the CVD and are important risk factors for other CVDs.^296,297^ The most common causes of CVD-related deaths include ischemic heart disease (IHD), atrial fibrillation (AF), cardiomyopathy, hypertensive heart disease, endocarditis, myocarditis, and others. The ischemic heart disease (IHD), also known as coronary heart disease (CHD), and stroke are the major culprits.^297^ Multiple studies revealed that RNA modifications and their regulators play essential roles in CVD (Table 1). This section mainly focuses on the heart (Fig. 8), while some vascular diseases, such as stroke and peripheral arterial disease (PAD), are not discussed.

**Table 1:** Regulators of RNA modifications in CVD

RNA modification	Regulator	Disease	Expression in disease	Mechanisms/functions	Refs.
m^6^A	METTL3 (writer)	Atherosclerosis	Upregulated	METTL3-m^6^A promoting *NLRP1* mRNA stability by YTHDF1, *KLF4* mRNA degradation by YTHDF2, and adhesion of monocytes to ECs	^303^
	METTL3 (writer)	MI	Upregulated	METTL3-m^6^A-HNRNPA2B1 promoting miR-503 biogenesis and CM apoptosis	^315^
	METTL3 (writer)	Cardiac I/R injury	Upregulated	METTL3-m^6^A-HNRNPD promoting *TFEB* mRNA stability, with impaired autophagy and promoted apoptosis in CMs	^315^
	METTL3 (writer)	Cardiac hypertrophy and HF	Cardiac-specific cKO	Resulting in HF or aggravating stress-induced HF	^331^
	METTL14 (writer)	Atherosclerosis	Upregulated	METTL14-m^6^A-YTHDF1 promoting the of *FOXO1* mRNA translation and activating transcription of VCAM-1 and ICAM-1; METTL14-m^6^A promoting *MyD88* mRNA stability and inflammation in macrophages	^304,305^
	METTL14 (writer)	Diabetic cardiomyopathy	Upregulated	METTL14-m^6^A-YTHDF2-lncRNA *TINCR* promoting CM pyroptosis via the NLRP3-caspase-1 pathway	^324^
	METTL14 (writer)	Doxorubicin-induced cardiomyopathy	Upregulated	METTL14-m^6^A-IGF2BP1-lncRNA KCNQ1OT1 promoting CM ferroptosis	^327^
	FTO (eraser)	MI	Downregulated	FTO-m^6^A-*ATP2A2* mRNA increasing *ATP2A2* mRNA levels and affecting sarcomere contraction	^313^
	FTO (eraser)	Cardiac hypertrophy and HF	Cardiac-specific cKO	deteriorating transverse aortic constriction surgery-induced HFrEF	^329^
	ALKBH5 (eraser)	MI	Downregulated	ALKBH5-m^6^A-YTHDF1-YAP inhibiting CM proliferation	^313^
	YTHDC1 (reader)	DCM	Cardiac-specific cKO	resulting in aberrant splicing of m^6^A-modified *TTN*	^324^
	IGF2BP2 (reader)	Diabetic cardiomyopathy	Downregulated	IGF2BP2 promoting m^6^A-*p53* mRNA stability	^325^
	YTHDF2 (reader)	Cardiac hypertrophy and HF	Upregulated	YTHDF2 promoting m^6^A-*MYH7* mRNA stability	^333^
A-to-I editing	ADAR1 (writer)	Atherosclerosis	Upregulated	ADAR1-editing-*CTSS* mRNA promoting HuR stability; ADAR1-editing-lncRNA NEAT1 promoting VCAM-1 and ICAM-1 expression	^231,306^
	ADAR1 (writer)	DCM	Cardiac-specific cKO	Resulting in autoinflammatory	^246^
	ADAR2 (writer)	Hypertension	Downregulated	Impairing *FLNA* pre-mRNA editing and affecting smooth muscle contraction	^300^
	ADAR2 (writer)	Cardiac hypertrophy and HF	Downregulated	Increasing circRNA formation	^227^
ac^4^C	NAT10 (writer)	MI	Upregulated	NAT10-ac^4^C-*TFEC* mRNA promoting CM apoptosis with deteriorated cardiac function	^312^
τm^5^U-tRNA U34	GTPBP3 or MTO1 (writers)	HCM	Mutation	Resulting in mitochondrial dysfunction	^289,290,321^

*CVD* cardiovascular disease, *CHD* coronary heart disease, *ECs* endothelial cells, *MI* myocardial infarction, *I/R injury* ischemia-reperfusion injury, *CM* cardiomyocyte, *HCM* hypertrophic cardiomyopathy, *DCM* dilated cardiomyopathy, *HF* heart failure, *cKO* conditional knock-out

### Hypertension

Hypertension is the leading risk factor for CVD globally, but it is preventable.^296^ The occurrence of hypertension is strongly linked to genetic and environmental factors. The regulators of blood pressure (BP) are diverse and very complex, involving the renin-angiotensin-aldosterone system (RAAS), the sympathetic nervous system (SNS), the immune system, and oxidative stress,^298^ and regulate the function of vascular smooth muscle cells (VSCMs) together. ADAR2 is reported to be primarily responsible for *FLNA* editing in the cardiovascular system, where it regulates smooth muscle contraction. Mice with impaired *FLNA* pre-mRNA editing showed diastolic hypertension.^299^ Common genetic variants of FTO are reportedly associated with BP regulation in patients with hypertension.^300^ However, it remains unclear whether it acts in an m^6^A-dependent mechanism. Previous studies indirectly demonstrated that RNA modification may be involved in hypertension development. For example, HuR is a key executor for biological functions of m^6^A, uridylation and A-to-I RNA editing by interacting with modification sites or “readers”. HuR is reduced in the aorta of patients with hypertension, and specific *HuR*-cKO mice develop hypertension and cardiac hypertrophy.^301^ HuR reportedly binds to AREs in caveolin-1 mRNA and soluble guanylyl cyclase (sGC) mRNA and regulates BP.^301,302^

### Atherosclerosis and coronary heart disease

Atherosclerosis is an inflammatory disease characterized by the accumulation of fat and fiber in the wall arteries, known as atherosclerotic plaque. Fibrosis and calcification occur in the coronary, leading to plaque bleeding, rupture, and thrombosis, which impedes blood flow and ultimately results in CHD. CHD mainly includes acute myocardial infarction (AMI), chronic stable angina pectoris, chronic CHD, and heart failure (HF) due to CHD.^295^ The m^6^A modification involves inflammatory cascades in extravascular sites and vascular endothelial cells (ECs), initiating and progressing atherosclerosis. The m^6^A “writers” METTL3 and METTL14 are significantly upregulated in atherosclerosis models and patients with CHD.^303–305^ Moreover, m^6^A promotes the adhesion of monocytes to ECs. Mechanistically, Chien et al.^303^ found that in an oscillatory shear (OS) stress-induced EC model, increased METTL3 expression promotes the stability of *m*^*6*^*A-NLRP1* mRNA by YTHDF1, whereas it increases the degradation of *m*^*6*^*A-KLF4* mRNA by YTHDF2. Furthermore, METTL14 mediates m^6^A modification of the transcription factor *FOXO1* mRNA in TNF-α-induced ECs, and promotes *FOXO1* mRNA translation by YTHDF1, thus activating VCAM-1 and ICAM-1transcription.^304^ The expression of VCAM-1 and ICAM-1 can also be regulated by lncRNA NEAT1 that is stabilized by the AUF1 (an RBP) in an ADAR1 mediated A-to-I RNA editing-dependent manner.^306^ However, the underlying mechanism is unclear. Macrophage inflammation is also regulated by m^6^A. *METTL14*-KD significantly reduces the stability of *MyD88* mRNA in macrophages, which promotes macrophage M2 polarization and inhibits macrophage migration and adhesion by inhibiting NF-κB/IL-6 signaling.^305^ METTL3-m^6^A-YTHDF2 positively regulates oxidized low-density lipoprotein (oxLDL)-induced inflammation of macrophage and monocyte.^307,308^ ADAR1 and METTL3/14 also affect angiogenesis and vascular or valve calcification.^231,309,310^

In addition to inflammatory response and vascular function, RNA modification regulates cardiac function in CHD. For example, decreased expression of CUGBP1 in AMI models is attributed to cytoplasmic HuR re-localization and interaction with AREs in CUGBP1 3′UTR.^214^ CUGBP1 overexpression improves cardiac function in MI mice by modulating VEGF-A. PIWI-interacting RNA (piRNA) HAAPIR directly interacts with NAT10 and promotes CM apoptosis by enhancing ac^4^C acetylation of transcription factor EC (TFEC), which results in deteriorated cardiac function in MI.^311^ m^6^A levels increase with downregulated FTO expression in mammalian HF and CMs suffering from hypoxia, rather than cardiac fibroblasts (CFs) or ECs.^312^ FTO overexpression improves sarcomere contraction by regulating intracellular Ca^2+^ and cardiac function after MI, due to FTO-mediated demethylation of *ATP2A2* mRNA (coding SERCA2a protein) in response to increased *ATP2A2* mRNA levels. Similarly, ALKBH5 overexpression alleviates cardiac function post-MI and promotes CM proliferation via the m^6^A-YTHDF1-YAP axis.^313^ Furthermore, METTL3-m^6^A-HNRNPA2B1 promotes the biogenesis of extracellular vesicle (EV)-encapsulated miR-503 derived from ECs in AMI, which promotes CM apoptosis and cardiac dysfunction.^314^ Ischemia-reperfusion (I/R) injury is a severe cardiac ischemia complication resulting in CHD deterioration. The up-regulated METTL3 in the cardiac I/R injury model facilitates the binding of HNRNPD to m^6^A*-TFEB* mRNA with improved stability, impairs autophagy, and promotes apoptosis in CMs.^315^ Moreover, TFEB activates ALKBH5 transcription and inhibits METTL3 stability, thus forming a negative feedback loop. Additionally, the cardiac-specific *METTL14*-cKO alleviates I/R injury and cardiac dysfunction in mouse hearts with decreased expression of HF markers such as ANP, BNP, and β-MHC.^316^

### Cardiomyopathy

Cardiomyopathy can be classified into two types: primary and secondary. Primary cardiomyopathy mainly includes hypertrophic cardiomyopathy (HCM), arrhythmogenic right ventricular cardiomyopathy (ARVC), dilated cardiomyopathy (DCM), restrictive cardiomyopathy (RCM) and myocarditis.^317^ Multiple genetic alterations that cause primary cardiomyopathy are identified and validated. Nearly 70% of patients with HCM have mutations in β-myosin heavy chain (*MYH7*) and myosin binding protein C (*MYBPC3*).^318^ Other related genes including troponin T (*TNNT2*), α-myosin heavy chain (*MYH6*), titin (*TTN*), muscle LIM protein (*CSRP3*), telethonin (*TCAP*), vinculin (*VCL*), and junctophilin 2 (*JPH2*), are also discovered. *TTN*, *MYH7*, and *TNNT2* mutations also lead to DCM and RCM.^319,320^ Other DCM-related genes include *LMNA*, *RBM20*, and *BAG3*. *GTPBP3* or *MTO1* mutation-mediated impaired τm^5^U formation on mitochondrial tRNAs and mitochondrial dysfunction results in HCM.^289,290,321^ Cardiac-specific *PTCD1*-cKO causes DCM, because PTCD1 is essential for the Ψ on 16S rRNA at position 2509 and the stability of 16S rRNA, which affects the assembly of LSU, the mitochondrial function, and the following mTOR signaling.^322^ Gao et al.^323^ observed that cardiac-specific *YTHDC1*-cKO leads to DCM. Moreover, the YTHDC1 deletion results in an aberrant splicing of the m^6^A-modified *TTN*, with an increased ratio of N2BA:N2B isoforms. Gonzalez and colleagues^246^ reported that cardiac-specific *ADAR1*-cKO causes autoinflammatory myocarditis, eventually leading to DCM in mice. Furthermore, *IFIH1* (coding MDA5) deficiency protects against DCM and HF by inhibiting the autoinflammatory processes caused by the absence of ADAR1-mediated RNA editing.

The etiology of secondary cardiomyopathy is usually acquired and includes diabetes, sepsis, and cardiotoxicity. In diabetic cardiomyopathy, METTL14 is down-regulated and mediates CM pyroptosis via the NLRP3-caspase-1 pathway.^324^ Functionally, METTL14-mediated hypermethylation of lncRNA TINCR promotes TINCR decay through YTHDF2, which weakens the inhibition of TINCR on the target NLRP3. LncRNA *Airn* alleviates cardiac fibrosis in diabetic cardiomyopathy by inhibiting ubiquitination degradation of IMP2 (encoded by *IGF2BP2*) and stabilizing *p53* mRNA in an m^6^A-dependent manner.^325^ METTL14 seems to be a risk factor in doxorubicin (DOX)-induced cardiomyopathy by promoting ferroptosis.^326^

### Cardiac hypertrophy and heat failure

Cardiac hypertrophy, both physiological and pathological, is the enlargement of the heart and CMs, due to the adaptation to cardiac stress.^327^ It is reported that in exercise-induced physiological cardiac hypertrophy, the total mRNA m^6^A levels in the heart are significantly reduced, possibly due to the METTL14 down-regulation.^316^ Pathological cardiac hypertrophy is a major contributor to HF with preserved ejection fraction (HFpEF) or reduced ejection fraction (HFrEF), occurring in various cardiac diseases. Cardiac-specific *FTO*-cKO deteriorates transverse aortic constriction (TAC) surgery-induced HFrEF in mice.^328^ In turn, overexpression of FTO attenuates TAC-induced cardiac hypertrophy and HF.^329^ Dorn et al.^330^ found that METTL3 overexpression induces a compensatory cardiac hypertrophy phenotype without pathological changes, even under stress stimuli such as pressure overload. Cardiac-specific *METTL3*-cKO results in HF or aggravates stress-induced HF in mice. The piRNA *CHAPIR* is identified as the upstream of METTL3.^331^ *CHAPIR* regulates cardiac hypertrophy by interacting with METTL3 and inhibiting its methylase activity, leading to the hypomethylation of the ADP-ribosyltransferase *Parp10* mRNA and the inhibition of YTHDF2-mediated degradation. Moreover, *MYH7*, a marker of cardiac hypertrophy, is also a target of YTHDF2. YTHDF2 is up-regulated in HF and promotes the *MYH7* stability in an m^6^A-dependent manner.^332^ Based on the sequencing technique, Kokot et al.^227^ explored the regulation of circRNAs by A-to-I RNA editing in human failing hearts. Their results showed that HF leads to decreased RNA editing with reduced ADAR2 protein and increased circRNA formation.

### Others

Pulmonary hypertension (PH) can be divided into five groups. Group 2 PH is caused by left heart disease, which is typically complicated in more than 50% of patients with HFpEF or HFrEF.^333^ PH is characterized by the proliferation and apoptosis-resistance of pulmonary artery smooth muscle cells (PASMCs).^334^ This phenotypic switch leads to pulmonary vascular remodeling (PVR). In PH, increased total RNA m^6^A levels are accompanied by elevated expression of some “writers” and “readers”, such as METTL3/14 and YTHDF1/2.^335,336^ In these cases, METTL3/14 catalyzes the methylation of downstream genes associated with proliferation, while YTHDF1/2 promotes their translation and degradation. For example, the translation of *MAGED1* is up-regulated by the METTL3-m^6^A-YTHDF1 axis.^335^ Functionally, *MAGED1*-KO inhibits hypoxia-induced PASMC proliferation and improves PVR and cardiac function in mice exposed to SU5416/hypoxia (Su/Hx). Chronic inflammation also contributes to PVR. YTHDF2 reportedly up-regulates in pulmonary macrophages of PH. YTHDF2 promotes PASMC proliferation by enhancing the m^6^A-*HMOX1* degradation, which activates macrophage, inflammatory response, and oxidative stress. Myeloid-specific *YTHDF2*-cKO inhibits macrophage activation, PVR, and cardiac dysfunction in Su/Hx-induced PH mice.^337^

Severe arrhythmias, such as ventricular tachycardia (VT), ventricular fibrillation (VF) and AF, can lead to death. Among them, ventricular arrhythmias (VAs), including VT and VF, are the leading causes of sudden cardiac death (SCD), while AF is usually closely related to stroke and HF.^338^ Patients with MI or HF are often observed with increased sympathetic nerve activity which can lead to frequent arrhythmias.^339^ In MI animal models, the expression levels of METTL3 are upregulated in the heart and paraventricular nucleus (PVN), the cardiovascular regulatory region in the brain, and are responsible for receiving cardiac reflex information and regulating sympathetic nerve activity for feedback.^340,341^ It is associated with the activation of the NF-κB pathway, mediated by TRAF6 and TLR4, respectively. Conversely, METTL3 silencing inhibits sympathetic remodeling and hyperactivity, which is beneficial in attenuating MI and improving cardiac function. Similarly, elevated METTL3 expression also regulates cardiac fibrosis and AF in an m^6^A-dependent manner.^342^

Aortic dissection (AD) is a dangerous and fatal disease. Hypertension and atherosclerosis are major risk factors for AD.^343^ The loss of aortic smooth muscle cells (ASMCs) is one of the characteristics of AD. Li et al.^344^ reported that METTL3/14 expression is significantly increased with reduced FTO expression in the aortas of patients with Stanford type A AD (TAAD) compared to non-AD aortas. METTL3 may promote ferroptosis in human ASMCs by facilitating the degradation of m^6^A-modified *SLC7A11* and *FSP1* mRNA. However, more studies are required to confirm the functions and specific molecular mechanisms of RNA m^6^A modification in AD.

## RNA modification-based therapeutics for cardiovascular disease

Regulators of RNA modification are involved in the initiation and development of CVD by affecting various phenotypes of different cells in the cardiovascular system and associated signaling pathways. The principles of RNA modification-based therapeutic strategies for CVD mainly include: inhibiting inflammatory response, reducing cell loss, attenuating damage, and promoting regeneration or alleviating remodeling. Moreover, RNA modification-based therapeutic strategies may act through multiple mechanisms and lead to better outcomes (Table 2).

**Table 2:** RNA modification based therapeutic strategies

RNA modification	Cardiovascular disease	Intervention	Functions/mechanisms	Refs.
m^6^A	Heart injury	AAV9-*shMETTL3* injection	Promoting CM proliferation and heart regeneration	^353^
	Cardiac hypertrophy and HF	AAV9-*shMETTL3* or AAV9-FTO-overexpression injection	Inhibiting cardiac dysfunction and remodeling	^330,363^
	Cardiac hypertrophy and HF	LV-*shMETTL3* or LV-*shYTHDF2* injection	Alleviating cardiac fibrosis and function	^325,342,363^
	Cardiac hypertrophy	Maslinic acid (MA)	Decreasing total m^6^A levels and METTL3 expression	^367^
	Cardiac hypertrophy	Tanshinone IIA (Tan IIA)	Inhibiting ALKBH5-mediated demethylation of galectin-3	^369^
	Myocardial I/R injury	LV-*shMETTL3* injection	Inhibiting pyroptosis	^361^
	MI	A nanomedicine HSSS-I with ALKBH5 inhibitor IOX1	Inhibiting apoptosis	^358^
	CAD	arsenic trioxide (ATO) for drug-eluting stent	Promoting ASMC apoptosis	^365^
	HF	Exercise	Inhibiting CM apoptosis	^359^
	Atherosclerosis	Exercise	Inhibiting EC pyroptosis	^359^
	High-fat diet-induced obesity cardiomyopathy	Intermittent fasting	Inhibiting CM apoptosis	^361^
m^5^C	Cardiac OFT malformations	*NSUN5* mutation	Decreasing cell proliferation	^346^
RNA editing	MI	Exercise	Inhibiting CM apoptosis	^230^

*m*^*6*^*A* N^6^-methyladenosine, *m*^*5*^*C* 5-methylcytosine, *AAV9* adeno-associated virus 9, *CM* cardiomyocyte, *LV* lentivirus, *HF* heart failure, *MI* myocardial infarction, *I/R* injury ischemia reperfusion injury, *CAD* coronary artery disease, *EC* endothelial cell, *OFT* outflow tract

### Cardiac development and regeneration

The regenerative capacity of the adult mammalian heart is very limited. The loss of CMs can be permanent and difficult to repair. Thus, stimulating CM dedifferentiation and proliferation provides a promising perspective for cardiac regeneration strategies.^345^ NSUN5-mediated RNA m^5^C modification is required for the translation of *TPM1*, a cardiac development-related gene.^346^ *NSUN5* mutation may cause decreased cell proliferation in the cardiac outflow tract (OFT) and OFT malformations in mice. Chen and colleagues^223^ explored the role of A-to-I RNA editing in the differentiation of human CMs. Their results showed that the proportion of editing sites in 3′UTRs decreases gradually and conversely, and the proportion of editing sites in introns increases during the process from undifferentiated cells to differentiated CMs. ADAR1 is essential for embryonic cardiac growth and development. Moore et al.^347^ observed that *ADAR1*-cKO results in apoptosis, reduced CM proliferation, and embryonic death. Han and colleagues^348^ revealed that total m^6^A levels increase with reduced ALKBH5 expression during the differentiation of mesoderm cells (MESs) into CMs. Consistently, Yang et al.^349^ found that the methylation peak in the heart of mice increases after birth and may plateau after 7 days. Furthermore, YTHDF1 and YTHDF3 are downregulated during the differentiation of embryonic stem cells (ESCs) into CMs.^350^ However, YTHDF1 and YTHDF3 regulate the expression of cardiac genes in CMs in opposite ways. Compared to embryonic hearts, m^6^A modification increases the expression of miR-133a and miR-499 in postnatal mouse hearts, thereby enhancing the inhibition of target genes involved in cardiac development.^351^ The cell cycle regulators, such as cyclins, cell cycle-independent kinases (CDKs) and proto-oncoproteins, promote CM proliferation.^345^ ALKBH5 promotes CM proliferation by positively regulating YAP expression, whereas METTL3 inhibits YAP expression via a negative regulation. Specifically, ALKBH5 is a demethylase of YAP and promotes its translation in a YTHDF1-dependent manner. Moreover, METTL3-mediated methylation of pri-miR-143 facilitates its maturation and inhibits the expression of YAP.^313,352^ In vivo injection of adeno-associated virus 9 (AAV9)-*shMETTL3* promotes CM proliferation and heart regeneration in the injured heart but has no significant effect on the heart without injury.^353^ In addition, METTL14 is also thought to be beneficial to CM apoptosis and detrimental to CM proliferation by promoting the interaction of YTHDF1 with m^6^A-*PHLPP2* mRNA with improved translation.^316^ However, ALKBH5 negatively regulates angiogenesis in cardiac microvascular endothelial cells (CMECs), detrimental to blood flow recovery in ischemic diseases.^354^

### Preventing cell loss and remodeling

Cell loss is one of the critical pathologic bases in various CVDs. For example, the loss of CMs leads to fibrosis, decreased cardiac contraction, and pathological cardiac dilatation, known as pathological cardiac remodeling, which subsequently deteriorates these processes and ultimately results in HF.^355^ The leading causes of cell loss in CVDs include apoptosis, necrosis, ferroptosis, and pyroptosis.^356^ Cheng et al.^357^ designed a nanomedicine to ameliorate myocardial I/R injury based on the ALKBH5 inhibitor IOX1, called HSSS-I. One advantage of HSSS-I is that it can actively target the infarct area and inhibit apoptosis. In addition, exercise may significantly improve cardiac function by attenuating RNA editing and m^6^A-mediated CM apoptosis.^229,358^ Exercise also alleviates atherosclerosis by inhibiting m^6^A-regulated EC pyroptosis.^359^ Intermittent fasting (IF) may also inhibit CM apoptosis in high-fat diet-induced obesity cardiomyopathy through a similar mechanism.^360^ Although exercise and IF are unlikely to be treated clinically, they may benefit patients as health management strategies. The tail vein injection of lentivirus (LV)-*shMETTL3* significantly inhibits pyroptosis in myocardial I/R injury mouse models.^361^ Liproxstatin-1, an inhibitor of ferroptosis, inhibits β-aminopropionitrile (BAPN)-induced medial degeneration and fragmentation of elastin in the aorta, significantly reducing morbidity and mortality.^344^ Similarly, MCC950, a pyroptosis inhibitor, partially attenuates cardiac injury in diabetic cardiomyopathy.^324^ It is unclear whether these inhibitors directly affect RNA modification or may act on the modified or downstream targets. We look forward to more studies to discover agents directly targeting RNA modification regulators and explore mechanisms and therapeutic effects. Additionally, AAV9-*shMETTL3* or AAV9-*FTO*-overexpression via intravenous injection reportedly inhibits cardiac dysfunction and remodeling in mouse models of myocardial hypertrophy.^329,362^

### Others

Cell proliferation is not always beneficial for CVD in all cases. For example, m^6^A-mediated aberrant proliferation of CFs results in cardiac fibrosis. Injection of LV-*shMETTL3* or LV-*shYTHDF2* significantly alleviates isoproterenol (ISO)-induced cardiac fibrosis in mice.^325,342,363^ Preventing ASMC proliferation and neointima formation after stenting or coronary artery bypass grafting improves patient outcomes. METTL3-mediated m^6^A modification promotes autophagosome formation to suppress ASMC proliferation, suggesting METTL3 as a potential therapeutic target.^364^ A clinical trial showed that arsenic trioxide (ATO) could be applied to drug-eluting stents with comparable efficacy and safety to traditional sirolimus-eluting ones.^365^ Yu et al.^366^ demonstrated that ATO selectively promotes apoptosis in ASMC by regulating m^6^A modification.

The application and mechanism of extracts from some natural plants or traditional Chinese medicines for CVD based on RNA modification have also received much attention. Maslinic acid (MA), a pentacyclic triterpenoid rich in olive pericarp, inhibits cardiac hypertrophy by decreasing total m^6^A levels and METTL3 expression.^367^ However, this mechanism has not been clarified. Tanshinone IIA (Tan IIA), an extract of traditional Chinese medicine *Salvia miltiorrhiza* Bunge, inhibits ALKBH5-mediated m^6^A modification of galectin-3, and may have a similar effect for CVD.^368^

## Conclusions and perspectives

Post-transcriptional modifications widely exist in transcripts, regulating gene expression, affecting cell differentiation and organ development, and determining cell fate. These chemical processes are dynamic based on the roles of “writers” and “erasers”, which are reportedly reversible (such as m^1^A and m^6^A) or transformed (for example, from m^5^C to hm^5^C), and thus impact RNA metabolisms and biological functions. The dynamic feature is also reflected in changes in the RNA modification distribution and the regulator expression during cell growth, differentiation, and organ development. It suggests that changes in chemical modification may allow cells to adapt to internal or external stimuli. For example, serum starvation, glucose deprivation, hypoxia and pressure-overload stimulation alter the levels of m^1^A, m^6^A, and pseudouridylation and the expression of regulators.^26,142,146,312,316^ Cellular and tissue damage induced by these stimuli is a crucial therapeutic mechanism in CVDs, including MI and myocardial I/R injury. Chemical modification promotes or repeals the recruitment or accessibility of regulatory elements, especially RBPs, directly or with the synergy of “readers”, thus controlling downstream signaling, such as inflammatory response, an initial event or exacerbating factor of CVDs. These suggest that RNA modification can either respond to stimuli or lead to changes in the microenvironment, and there may be a complex cross-talk.

Despite tremendous progress, there are still many unknown or controversial issues. First, RNA modification regulators prefer substrates, sites, and functions. How regulators choose substrates and specific sites, how substrates are altered with condition changes, and how “readers” perform one of their functions need to be elucidated. Cell- or tissue-specific RNA modification patterns or regulators are observed. YTHDF1-3 are characterized as m^6^A “readers”. They are also candidates for m^1^A “readers” in Hela cell,^24,369,370^ while only YTHDF3 is reconfirmed in HEK293T and Raw264.7 cells,^371^ suggesting the potential cell specificity. However, false positive results cannot be excluded either. For instance, YTHDF2 reportedly mediates m^1^A-modified RNA degradation in an m^6^A-dependent manner rather than acts as an m^1^A “reader” in Hela cell.^372^ The direct binding of HRSP12 to the m^1^A motif promotes the binding of YTHDF2 to the m^6^A site, resulting in RNase P/MRP complex-mediated endoribonucleolytic cleavage. Cross-talk between chemical modification makes the regulatory mechanism even more intricate. We propose three types of cross-talk: 1) a transcript can be modified by different modifications with synergy or rejection effect by different regulators or a shared regulator (for example, m^1^A/m^6^A “eraser” FTO and m^6^A/m^5^C “reader” YTHDF2); 2) regulators of one modification are modified by another modification; 3) There are direct interactions between regulators of different modifications. Both m^6^A and A-to-I RNA editing are widely present in RNA, and developing techniques for detecting RNA modification may benefit CVD treatments. Although the mechanisms of m^5^C DNA methylation in myocardial infarction and dilated cardiomyopathy have been reported,^373,374^ there are few studies of m^5^C on RNA in CVD. Several studies have explored the role of RBPs in regulating the expression of genes enriched with AREs in CVD. However, the role of uridylyl transferases in the dynamic regulation of uridylation in CVD remains unclear. Effective prevention and intervention of CVD remains challenging; hence CVD continues to be a leading cause of death worldwide. The introduction of precision medicine and its application in CVD is expected to improve the prognosis of patients, based on the development of pan-omics, including genomics, transcriptomics, epigenomics, and proteomics.^375^ Fortunately, RNA modification-based therapeutic strategies have shown certain effects on CVD. Gene therapy using viral vectors to transfer genes to CVD is promising since several clinical trials have demonstrated its safety and efficacy.^376^ RNA modification-based gene therapy shows therapeutic effects in animal CVD models through various mechanisms. For example, interference with AAV9/LV-*shMETTL3* promotes cardiomyocyte proliferation, inhibits cardiomyocyte death, and attenuates cardiac remodeling.^330,342,363^ It should be noted that the role of a regulator may be totally reversed in different cell types, and this could also be a potential problem for gene therapy, indicating the need for specific cell targeting to provide high therapeutic efficacy and avoid adverse effects. Nanotechnology could be a viable solution.^377^ Nanomedicine has the advantage of precise targeting with reduced side effects, facilitating combination therapy by a nanoplatform, and ultimately delivering better therapeutic effects at lower doses. Moreover, the potential of some FDA-approved drugs and compounds derived from natural plants or traditional Chinese herbal medicines for treating CVD should not be overlooked. They may be more accessible to clinical applications with potentially higher cost-effectiveness than nanomedicine, which remains an open clinical translation challenge, such as pharmaceutical metabolism in vivo. In addition, we expect to see the identification of more molecular inhibitors or activators targeting RNA modification regulators, particularly for CVD.

### Supplementary information

Supplementary Table1
