# Supplementary material for: RNA modification in cardiovascular disease: implications for therapeutic interventions
Source: Signal Transduct Target Ther. 2023 Oct 27;8:412. doi: 10.1038/s41392-023-01638-7 (PMC10603151; doi:10.1038/s41392-023-01638-7)
Supplement: Supplementary file 1 — Supplementary Table1 [file 41392_2023_1638_MOESM1_ESM.docx]

**SupplementaryTable 1. Clinical implication of RNA modification in human diseases**

| **Clinical implication** | **RNA modification** | **Biomarker** | **Disease** | **Expression/Function** | **Ref.** |
| --- | --- | --- | --- | --- | --- |
| **Diagnostic biomarker** | **m^6^A** | m^6^A RNA levels | Gastric cancer | Up-regulated in peripheral blood | 1 |
|  |  |  | Type 2 diabetes mellitus | Down-regulated in peripheral blood | 2 |
| **Prognostic biomarker** | **m^6^A** | METTL3 | Endometrial cancer | Low expression associated with poor prognosis | 3 |
|  |  |  | Various cancers | High expression associated with poor prognosis | 4-9 |
|  |  | METTL14 | Various cancers | Low expression associated with poor prognosis | 10-12 |
|  |  | WTAP | Bladder cancer | High expression associated with poor prognosis | 13 |
|  |  | VIRMA | Lung adenocarcinoma | High expression associated with poor prognosis | 14 |
|  |  | ALKBH5 | Colorectal cancer, acute myeloid leukemia | High expression associated with poor prognosis | 15,16 |
|  |  |  | Hepatocellular carcinoma, pancreatic cancer | Low expression associated with poor prognosis | 17,18 |
|  |  | FTO | Bladder cancer, breast cancer | High expression associated with poor prognosis | 19,20 |
|  |  | YTHDF1 | Hepatocellular carcinoma | High expression associated with poor prognosis | 21 |
|  |  | YTHDF2 | Prostate cancer | High expression associated with poor prognosis | 8 |
|  |  | IGF2BP2 | Head and neck squamous carcinoma, colorectal cancer | High expression associated with poor prognosis | 22,23 |
|  | **m^5^C** | NSUN2 | Esophageal squamous cell carcinoma | High expression associated with poor prognosis | 24 |
|  | **ac^4^C** | NAT10 | Gastric cancer, colorectal cancer | High expression associated with poor prognosis | 25,26 |
|  | **A-to-I editing** | ADAR1/2 | Hepatocellular carcinoma | High/Low expression associated with poor prognosis | 27 |
|  | **Pseudouridine** | PUS7 | Glioblastoma | High expression associated with poor prognosis | 28 |
| **Drug discovery** |  |  |  |  |  |
| **Inhibitor** |  |  |  |  |  |
| STM2457 | **m^6^A** | METTL3 | Myeloid leukemia | Inhibiting AML growth and promoting differentiation and apoptosis | 29 |
| ALK-04 | **m^6^A** | ALKBH5 | Melanoma | Enhancing the efficacy of anti-PD-1 therapy | 30 |
| IOX1 | **m^6^A** | ALKBH5 | Myocardial infarction | Inhibiting cardiomyocyte death | 31 |
| Meclofenamic acid | **m^6^A** | FTO | - | - | 32 |
| FB23/FB23-2 | **m^6^A** | FTO | Acute myeloid leukemia | Inhibiting AML progression | 33 |
| Tegaserod | **m^6^A** | YTHDF1 | Acute myeloid leukemia | Inhibiting cell viability | 34 |
| BTYNB | **m^6^A** | IGF2BP1 | Cancers | Inhibiting tumor growth | 35 |
| JX5 | **m^6^A** | IGF2BP2 | T-cell acute lymphoblastic leukemia | Providing an alternative γ-Secretase inhibitors therapy | 36 |
| Thiram | **m^1^A** | TRMT6 | Hepatocellular carcinoma | Inhibiting oncosphere formation | 37 |
| Azacytidine | **m^5^C** | DNMT2 | Cancers | - | 38 |
| Remodelin | **ac^4^C** | NAT10 | Gastric cancer, colorectal cancer, | Inhibiting tumor growth | 25,26 |
| Rebecsinib | **A-to-I editing** | ADAR1 | Leukemia | Inhibiting leukemia stem cell self-renewal | 39 |
| pyrazofurin | **Pseudouridine** | DKC1 | Colorectal cancer | Inhibiting tumor growth | 40 |
| **Nanomedicine** |  |  |  |  |  |
| HSSS-I | **m^6^A** | ALKBH5 | Myocardial infarction | Improving the cardiac function and decreasing the infarct size | 31 |
| Nb-S2A4 | **Uridylation** | TUT4 | - | - | 41 |
| **Other intervention** |  |  |  |  |  |
| Chemotherapy | **m^6^A** | METTL3 | Gastrointestinal stromal tumor | Promoting resistance to imatinib | 6 |
|  |  | METTL16 | Pancreatic ductal adenocarcinoma | Increasing sensitivity to gemcitabine | 42 |
|  |  | ZCCHC4 | Small-cell lung cancer | Increasing sensitivity to cisplatin | 43 |
|  |  | WTAP | Bladder cancer | Promoting resistance to cisplatin | 13 |
|  |  | ALKBH5 | Pancreatic ductal adenocarcinoma | Increasing sensitivity to gemcitabine | 44 |
|  |  | YTHDF2 | Intrahepatic cholangiocarcinoma | Promoting resistance to cisplatin | 45 |
|  |  | IGF2BP2 | Radioiodine-refractory papillary thyroid cancer | Promoting acquired resistance to tyrosine kinase inhibitor | 46 |
|  |  | IGF2BP3 | Laryngeal squamous cell carcinoma | Promoting resistance to cisplatin | 47 |
| Radiotherapy | **A-to-I editing** | ADAR1 | Melanoma | ADAR1-deficiency enhancing response to irradiation | 48 |
| Targeted therapy | **m^6^A** | METTL3 | Hepatocellular carcinoma | Promoting resistance to Lenvatinib | 49 |
|  | **m^7^G** | METTL1 | Hepatocellular carcinoma | Promoting resistance to Lenvatinib | 50 |
| Immunotherapy | **m^6^A** | METTL3 | Melanoma | METTL3 deficiency decreasing efficacy of anti-PD-1 therapy | 51 |
|  |  | ALKBH5 | Colorectal cancer and melanoma | ALKBH5 deficiency enhancing response to anti-PD-1 therapy | 30 |
|  |  | FTO | melanoma | FTO deficiency enhancing response to anti-PD-1 therapy | 52 |
|  |  | YTHDF1 | Colorectal cancer and melanoma | YTHDF1 deficiency enhancing response to anti-PD-1 therapy | 53 |
|  | **A-to-I editing** | ADAR1 | Melanoma | ADAR1 deficiency enhancing response to anti-PD-1 therapy | 48 |
| Gene therapy |  |  |  |  |  |
| Antisense oligonucleotides | **A-to-I editing** |  | - | - | 54 |

**Reference**

1 Ge, L. *et al.* Level of N6-Methyladenosine in Peripheral Blood RNA: A Novel Predictive Biomarker for Gastric Cancer. *Clin. Chem.* **66**, 342-351, (2020).

2 Shen, F. *et al.* Decreased N(6)-methyladenosine in peripheral blood RNA from diabetic patients is associated with FTO expression rather than ALKBH5. *J Clin Endocrinol Metab* **100**, E148-154, (2015).

3 Zhan, L. *et al.* METTL3 facilitates immunosurveillance by inhibiting YTHDF2-mediated NLRC5 mRNA degradation in endometrial cancer. *Biomark Res* **11**, 43, (2023).

4 Du, Q. Y. *et al.* METTL3 potentiates progression of cervical cancer by suppressing ER stress via regulating m6A modification of TXNDC5 mRNA. *Oncogene* **41**, 4420-4432, (2022).

5 Yue, B. *et al.* METTL3-mediated N6-methyladenosine modification is critical for epithelial-mesenchymal transition and metastasis of gastric cancer. *Mol. Cancer* **18**, 142, (2019).

6 Xu, K. *et al.* N(6)-methyladenosine modification regulates imatinib resistance of gastrointestinal stromal tumor by enhancing the expression of multidrug transporter MRP1. *Cancer Lett.* **530**, 85-99, (2022).

7 Li, T. *et al.* METTL3 facilitates tumor progression via an m(6)A-IGF2BP2-dependent mechanism in colorectal carcinoma. *Mol. Cancer* **18**, 112, (2019).

8 Li, J. *et al.* YTHDF2 mediates the mRNA degradation of the tumor suppressors to induce AKT phosphorylation in N6-methyladenosine-dependent way in prostate cancer. *Mol. Cancer* **19**, 152, (2020).

9 Liu, L. *et al.* METTL3 Promotes Tumorigenesis and Metastasis through BMI1 m(6)A Methylation in Oral Squamous Cell Carcinoma. *Mol. Ther.* **28**, 2177-2190, (2020).

10 Wang, M. *et al.* Upregulation of METTL14 mediates the elevation of PERP mRNA N(6) adenosine methylation promoting the growth and metastasis of pancreatic cancer. *Mol. Cancer* **19**, 130, (2020).

11 Chen, X. *et al.* METTL14-mediated N6-methyladenosine modification of SOX4 mRNA inhibits tumor metastasis in colorectal cancer. *Mol. Cancer* **19**, 106, (2020).

12 Yang, X. *et al.* METTL14 suppresses proliferation and metastasis of colorectal cancer by down-regulating oncogenic long non-coding RNA XIST. *Mol. Cancer* **19**, 46, (2020).

13 Wei, W. *et al.* Circ0008399 Interaction with WTAP Promotes Assembly and Activity of the m(6)A Methyltransferase Complex and Promotes Cisplatin Resistance in Bladder Cancer. *Cancer Res.* **81**, 6142-6156, (2021).

14 Zhang, C. *et al.* Gene amplification-driven RNA methyltransferase KIAA1429 promotes tumorigenesis by regulating BTG2 via m6A-YTHDF2-dependent in lung adenocarcinoma. *Cancer Commun (Lond)* **42**, 609-626, (2022).

15 Shen, D. *et al.* RNA demethylase ALKBH5 promotes colorectal cancer progression by posttranscriptional activation of RAB5A in an m6A-YTHDF2-dependent manner. *Clin Transl Med* **13**, e1279, (2023).

16 Li, R. *et al.* RNA demethylase ALKBH5 promotes tumorigenesis of t (8;21) acute myeloid leukemia via ITPA m6A modification. *Biomark Res* **11**, 30, (2023).

17 Chen, Y. *et al.* ALKBH5 suppresses malignancy of hepatocellular carcinoma via m(6)A-guided epigenetic inhibition of LYPD1. *Mol. Cancer* **19**, 123, (2020).

18 Guo, X. *et al.* RNA demethylase ALKBH5 prevents pancreatic cancer progression by posttranscriptional activation of PER1 in an m6A-YTHDF2-dependent manner. *Mol. Cancer* **19**, 91, (2020).

19 Tao, L. *et al.* FTO modifies the m6A level of MALAT and promotes bladder cancer progression. *Clin Transl Med* **11**, e310, (2021).

20 Niu, Y. *et al.* RNA N6-methyladenosine demethylase FTO promotes breast tumor progression through inhibiting BNIP3. *Mol. Cancer* **18**, 46, (2019).

21 Li, Q. *et al.* HIF-1α-induced expression of m6A reader YTHDF1 drives hypoxia-induced autophagy and malignancy of hepatocellular carcinoma by promoting ATG2A and ATG14 translation. *Signal Transduct Target Ther* **6**, 76, (2021).

22 Yu, D. *et al.* RNA N6-methyladenosine reader IGF2BP2 promotes lymphatic metastasis and epithelial-mesenchymal transition of head and neck squamous carcinoma cells via stabilizing slug mRNA in an m6A-dependent manner. *J. Exp. Clin. Cancer Res.* **41**, 6, (2022).

23 Lu, S. *et al.* N6-methyladenosine reader IMP2 stabilizes the ZFAS1/OLA1 axis and activates the Warburg effect: implication in colorectal cancer. *J Hematol Oncol* **14**, 188, (2021).

24 Su, J. *et al.* NSUN2-mediated RNA 5-methylcytosine promotes esophageal squamous cell carcinoma progression via LIN28B-dependent GRB2 mRNA stabilization. *Oncogene* **40**, 5814-5828, (2021).

25 Deng, M. *et al.* Helicobacter pylori-induced NAT10 stabilizes MDM2 mRNA via RNA acetylation to facilitate gastric cancer progression. *J. Exp. Clin. Cancer Res.* **42**, 9, (2023).

26 Jin, C. *et al.* Acetyltransferase NAT10 regulates the Wnt/β-catenin signaling pathway to promote colorectal cancer progression via ac(4)C acetylation of KIF23 mRNA. *J. Exp. Clin. Cancer Res.* **41**, 345, (2022).

27 Chan, T. H. *et al.* A disrupted RNA editing balance mediated by ADARs (Adenosine DeAminases that act on RNA) in human hepatocellular carcinoma. *Gut* **63**, 832-843, (2014).

28 Cui, Q. *et al.* Targeting PUS7 suppresses tRNA pseudouridylation and glioblastoma tumorigenesis. *Nat Cancer* **2**, 932-949, (2021).

29 Yankova, E. *et al.* Small-molecule inhibition of METTL3 as a strategy against myeloid leukaemia. *Nature* **593**, 597-601, (2021).

30 Li, N. *et al.* ALKBH5 regulates anti-PD-1 therapy response by modulating lactate and suppressive immune cell accumulation in tumor microenvironment. *Proc. Natl. Acad. Sci. U. S. A.* **117**, 20159-20170, (2020).

31 Cheng, P. *et al.* Amelioration of acute myocardial infarction injury through targeted ferritin nanocages loaded with an ALKBH5 inhibitor. *Acta Biomater.* **140**, 481-491, (2022).

32 Huang, Y. *et al.* Meclofenamic acid selectively inhibits FTO demethylation of m6A over ALKBH5. *Nucleic Acids Res.* **43**, 373-384, (2015).

33 Huang, Y. *et al.* Small-Molecule Targeting of Oncogenic FTO Demethylase in Acute Myeloid Leukemia. *Cancer Cell* **35**, 677-691.e610, (2019).

34 Hong, Y. G. *et al.* The RNA m6A Reader YTHDF1 Is Required for Acute Myeloid Leukemia Progression. *Cancer Res.* **83**, 845-860, (2023).

35 Müller, S. *et al.* The oncofetal RNA-binding protein IGF2BP1 is a druggable, post-transcriptional super-enhancer of E2F-driven gene expression in cancer. *Nucleic Acids Res.* **48**, 8576-8590, (2020).

36 Feng, P. *et al.* Inhibition of the m(6)A reader IGF2BP2 as a strategy against T-cell acute lymphoblastic leukemia. *Leukemia* **36**, 2180-2188, (2022).

37 Wang, Y. *et al.* N(1)-methyladenosine methylation in tRNA drives liver tumourigenesis by regulating cholesterol metabolism. *Nat. Commun.* **12**, 6314, (2021).

38 Schaefer, M., Hagemann, S., Hanna, K. & Lyko, F. Azacytidine inhibits RNA methylation at DNMT2 target sites in human cancer cell lines. *Cancer Res.* **69**, 8127-8132, (2009).

39 Crews, L. A. *et al.* Reversal of malignant ADAR1 splice isoform switching with Rebecsinib. *Cell Stem Cell* **30**, 250-263.e256, (2023).

40 Kan, G. *et al.* Dual Inhibition of DKC1 and MEK1/2 Synergistically Restrains the Growth of Colorectal Cancer Cells. *Advanced science (Weinheim, Baden-Wurttemberg, Germany)* **8**, 2004344, (2021).

41 Yu, C. *et al.* A nanobody targeting the LIN28:let-7 interaction fragment of TUT4 blocks uridylation of let-7. *Proc. Natl. Acad. Sci. U. S. A.* **117**, 4653-4663, (2020).

42 Zeng, X. *et al.* METTL16 antagonizes MRE11-mediated DNA end resection and confers synthetic lethality to PARP inhibition in pancreatic ductal adenocarcinoma. *Nat Cancer* **3**, 1088-1104, (2022).

43 Zhang, Z. *et al.* m(6)A regulators as predictive biomarkers for chemotherapy benefit and potential therapeutic targets for overcoming chemotherapy resistance in small-cell lung cancer. *J Hematol Oncol* **14**, 190, (2021).

44 Tang, B. *et al.* m(6)A demethylase ALKBH5 inhibits pancreatic cancer tumorigenesis by decreasing WIF-1 RNA methylation and mediating Wnt signaling. *Mol. Cancer* **19**, 3, (2020).

45 Huang, C. S. *et al.* YTHDF2 promotes intrahepatic cholangiocarcinoma progression and desensitises cisplatin treatment by increasing CDKN1B mRNA degradation. *Clin Transl Med* **12**, e848, (2022).

46 Sa, R. *et al.* IGF2BP2-dependent activation of ERBB2 signaling contributes to acquired resistance to tyrosine kinase inhibitor in differentiation therapy of radioiodine-refractory papillary thyroid cancer. *Cancer Lett.* **527**, 10-23, (2022).

47 Yang, L. *et al.* IGF2BP3 Regulates TMA7-mediated Autophagy and Cisplatin Resistance in Laryngeal Cancer via m6A RNA Methylation. *Int. J. Biol. Sci.* **19**, 1382-1400, (2023).

48 Ishizuka, J. J. *et al.* Loss of ADAR1 in tumours overcomes resistance to immune checkpoint blockade. *Nature* **565**, 43-48, (2019).

49 Wang, L. *et al.* METTL3-m(6)A-EGFR-axis drives lenvatinib resistance in hepatocellular carcinoma. *Cancer Lett.* **559**, 216122, (2023).

50 Huang, M. *et al.* METTL1-Mediated m7G tRNA Modification Promotes Lenvatinib Resistance in Hepatocellular Carcinoma. *Cancer Res.* **83**, 89-102, (2023).

51 Yin, H. *et al.* RNA m6A methylation orchestrates cancer growth and metastasis via macrophage reprogramming. *Nat. Commun.* **12**, 1394, (2021).

52 Yang, S. *et al.* m(6)A mRNA demethylase FTO regulates melanoma tumorigenicity and response to anti-PD-1 blockade. *Nat. Commun.* **10**, 2782, (2019).

53 Han, D. *et al.* Anti-tumour immunity controlled through mRNA m(6)A methylation and YTHDF1 in dendritic cells. *Nature* **566**, 270-274, (2019).

54 Tay, D. J. T. *et al.* Targeting RNA editing of antizyme inhibitor 1: A potential oligonucleotide-based antisense therapy for cancer. *Mol. Ther.* **29**, 3258-3273, (2021).
